# Growth hormone receptor antagonism with pegvisomant in insulin resistant non-diabetic men: A phase II pilot study

**DOI:** 10.12688/f1000research.11359.1

**Published:** 2017-05-03

**Authors:** Ada P. Lee, Kathleen Mulligan, Morris Schambelan, Elizabeth J. Murphy, Ethan J. Weiss

**Affiliations:** 1 Department of Medicine, University of California, San Francisco, San Francisco, CA, 94143, USA; 2 Division of Endocrinology, San Francisco General Hospital, San Francisco, CA, 94110, USA; 3 Division of Cardiology, University of California, San Francisco, San Francisco, CA, 94143, USA; 4 Cardiovascular Research Institute, University of California, San Francisco, San Francisco, CA, 94143, USA

**Keywords:** Insulin resistance, Prediabetes, Pegvisomant, Growth Hormone, Metabolism

## Abstract

*Background: *Growth hormone (GH) is known to affect insulin and glucose metabolism.  Blocking its effects in acromegalic patients improves diabetes and glucose metabolism. We aimed to determine the effect of pegvisomant, a GH receptor antagonist, on insulin resistance, endogenous glucose production (EGP) and lipolysis in insulin resistant non-diabetic men.

*Methods:* Four men between the ages of 18-62 with a BMI of 18-35kg/m ^2^, with insulin resistance as defined by a HOMA-IR > 2.77, were treated for four weeks with pegvisomant 20 mg daily.  Inpatient metabolic assessments were performed before and after treatment. The main outcome measurements were: change after pegvisomant therapy in insulin sensitivity as measured by hyperinsulinemic euglycemic clamp; and EGP and lipolysis assessed by stable isotope tracer techniques.

*Results: *Insulin like growth factor-1 (IGF-1) concentrations decreased from 134.0 ± 41.5 (mean ± SD) to 72.0 ± 11.7 ng/mL (p = 0.04) after 4 weeks of therapy. Whole body insulin sensitivity index (M/I 3.2 ± 1.3 *vs.* 3.4 ± 2.4; *P* = 0.82), as well as suppression of EGP (89.7 ± 26.9 *vs.* 83.5 ± 21.6%; p = 0.10) and Ra glycerol (59.4 ± 22.1% *vs.* 61.2 ± 14.4%; p = 0.67) during the clamp were not changed significantly with pegvisomant treatment.

*Conclusions:* Blockade of the GH receptor with pegvisomant for four weeks had no significant effect on insulin/glucose metabolism in a small phase II pilot study of non-diabetic insulin resistant participants without acromegaly.

## Introduction

The worldwide incidence of type 2 diabetes (T2DM) has increased dramatically ^1^. Insulin resistance (IR) plays a critical role in the pathogenesis of T2DM, but the mechanisms underlying insulin resistance in target tissues remain complex and unresolved ^2^. Insulin regulates the metabolism of glucose, lipids and proteins in multiple tissues, including liver, muscle, and fat ^3^. There are individuals who have been described as ‘fit and fat’ – insulin sensitive despite a high body mass index (BMI) ^4^. There are also well-known examples of individuals with insulin resistance despite a low BMI ^5^. This dissociation between total adiposity and insulin sensitivity is especially significant in growth hormone (GH) disorders. In acromegaly, a condition of GH excess, there is low body fat with insulin resistance, and in Laron’s syndrome, due to an inactivating mutation in the growth hormone receptor (GHR), there is high body fat with insulin sensitivity ^6^.

GH is a known regulator of lipid and carbohydrate metabolism. Excessive GH secretion in acromegaly can lead to insulin resistance and diabetes, while reducing overall fat mass. Pegvisomant is a specific and competitive antagonist for the GHR that effectively blocks GH signaling ^7^. Treatment of acromegaly, including medical treatment with pegvisomant, improves insulin sensitivity and glucose metabolism ^8, 9^. The reduction of GH signaling in mice by global disruption of *Ghr* also leads to improved insulin sensitivity, lower fasting glucose and insulin levels, and increased longevity despite an increase in body fat ^10^. Mice with global disruption of *Ghr* are also protected from high fat diet-induced changes in carbohydrate metabolism despite increased body fat ^10^. Similarly, humans with Laron’s syndrome are exquisitely insulin sensitive despite increased adiposity ^6, 11^. Compared to age and sex-matched relatives, people with inactivating mutations in *Ghr* are dramatically protected from diabetes over 22 years of follow up ^12^.

Intact GH signaling appears to play an important role in insulin action. There are very few studies of substrate metabolism in healthy subjects treated with pegvisomant ^13, 14^. In these studies, pegvisomant treatment was short, often single dose, and an effect on substrate metabolism and insulin resistance was not observed. There is a single small study that demonstrated an improvement in hepatic insulin sensitivity in patients with type 1 diabetes treated with pegvisomant ^15^. In this phase II pilot study, we sought to determine the effect of longer-term treatment with pegvisomant on insulin resistance in pre-diabetic men. We hypothesized that one month of pegvisomant treatment would improve whole body insulin sensitivity, as well as suppression of endogenous glucose production (EGP) and lipolysis, during a hyperinsulinemic euglycemic clamp.

## Methods

### Participants and study design

Four men, aged 52–57 years, with a BMI between 18–35 kg/m ^2^ and insulin resistance, defined as a HOMA-IR score >2.77 ^16^, were enrolled. The first subject was enrolled on March 3, 2014. The sample size was determined after a power analysis based on prior work and the effect size of the primary outcome. Participants were recruited through a combination of advertising, word of mouth, and doctor’s appointments. Participants were required to be on a stable medication regimen for any lipid disorders. Participants were excluded if they had type 1 or type 2 diabetes, fasting blood glucose >126 mg/dl, hemoglobin A1c >6.5, unstable hypertension, human immunodeficiency virus infection, hepatitis B or hepatitis C infection, evidence of chronic kidney disease, major gastrointestinal surgery, history of pancreatitis, pancreatic disease, liver or biliary disorders, or fasting plasma triglyceride >500 mg/dl. The study was completed in August 2015, and the final follow-up was completed in December 2015. The Committee on Human Research of the University of California, San Francisco approved the study (IRB Number: 13-10982; full protocol available as Supplementary File 1). Written, informed consent was obtained from each individual.

Participants were admitted to the Clinical Research Center (CRC) at San Francisco General Hospital the evening before baseline testing and consumed a controlled metabolic diet with fixed proportions of macronutrients (10 kcal/kg; 15% protein, 30% fat, 55% carbohydrate with <20% from simple sugars). All meals were prepared in the metabolic kitchen of the CRC under the supervision of CRC bionutritionists. In the morning, the participants underwent metabolic assessments, including measurements of body composition by dual-energy X-ray absorptiometry (DXA), resting energy expenditure by indirect calorimetry, and hyperinsulinemic-euglycemic clamp with stable isotope tracer infusions. After baseline testing, participants were discharged and self-administered pegvisomant 20 mg subcutaneously nightly, after supervised instruction from a CRC nurse. Participants were instructed to maintain their usual diets and activity levels and attended weekly follow up visits. During these visits, the participants had updated medical history and a brief physical exam, including weight and vital signs. All reported signs and symptoms were recorded. Safety laboratory studies (i.e. fasting glucose, lipids, electrolytes, and renal and hepatic function), as well as insulin, GH and IGF1 levels, were obtained. At these visits they also returned unused vials of drug and received their next week’s supply of drug and supplies. After four weeks of treatment, the inpatient metabolic assessments were repeated, as at baseline.

### Euglycemic, hyperinsulinemic clamp

Whole-body insulin sensitivity was measured by the euglycemic hyperinsulinemic clamp technique ^17^. After the baseline measurements were completed, insulin (Humulin®, Eli Lilly & Co., Indianapolis, IN, USA; 40 mU/m ^2^ min) was infused for 180 minutes and blood samples were collected at 5-minute intervals from a retrograde intravenous line placed in a hand that was warmed in a heated box at 50–55 ^o^C. Whole-blood glucose concentrations were determined by the glucose oxidase method (YSI Stat glucose analyzer, Yellow Springs, OH, USA). A variable infusion of 20% dextrose (labeled with [U- ^13^C] glucose, as described below) was adjusted to maintain blood glucose concentrations at 90 mg/dL. Blood samples were collected at 30-minute intervals during the final hour of the clamp and the serum was frozen and batched for measurement of insulin. Insulin sensitivity was calculated as a measure of whole-body glucose uptake during the final hour of the clamp (M) divided by steady-state serum insulin level (I) ^18^.

### Stable isotope tracer methods

EGP (R _a_ glucose) and whole body lipolysis (R _a_ glycerol) were measured under fasting conditions using primed constant infusions of [U- ^13^C] glucose (0.96 mg/kg/h, prime 0.096 mg/kg/min for 10 min) and [ ^2^H _5_]-glycerol (0.67 mg/kg/h, prime 0.067 mg/kg/min for 10 min) started at 430 h. Blood samples were obtained every 10 minutes between 0800 and 0830 h for steady-state fasting measurements. The isotope infusions continued, and a 180-minute euglycemic-hyperinsulinemic clamp was started at 0900 h, as described above. The constant glycerol infusion continued, while the enriched glucose became a variable infusion of 0.6% [U- ^13^C] glucose within the 20% dextrose infusion used for the clamp. During the final 30 minutes of the clamp (1130 to 1200 h), blood samples were collected every 10 minutes for determination of EGP and lipolysis under conditions of steady-state hyperinsulinemia.

Isotope enrichments were measured by Metabolic Solutions (Nashua, NH, USA). [ ^2^H _5_]-glycerol was determined by gas chromatography-mass spectrometry (GC-MS), using the trimethylsilyl (TMS) derivative ^19^. Plasma ^13^C _6_-glucose was determined using the aldonitrile penta-acetate derivative ^20^.

Ra glucose and Ra glycerol were calculated by the dilution technique using the average of the last 4 samples during the fasting state and during the clamp ^21^.

### Body composition

Total and regional fat and lean body mass were measured by DXA (Hologic, Marlborough, MA, USA) and subsequently analyzed using Apex 5.5™ software (Hologic, Marlborough, MA, USA) to provide estimates of visceral adipose tissue.

### Indirect calorimetry

Resting energy expenditure was measured by indirect calorimetry under fasting conditions and during the clamp using a Deltatrac II Metabolic Monitor (Sensormedics, Yorba Linda, CA, USA). Respiratory quotient (RQ), and index of substrate utilization, was calculated as carbon dioxide production divided by oxygen production rates.

### Other laboratory measures

Free insulin like growth factor (IGF)-1 and IGF-BP3 were determined by ELISA (GenWay Biotech, San Diego, CA, USA). The San Francisco General Hospital Clinical Laboratory measured total and high-density lipoprotein (HDL) cholesterol, fasting triglycerides (TG), and calculated low-density lipoprotein (LDL) cholesterol, serum insulin, and fasting serum glucose. Serum insulin was measured by chemiluminescent sandwich assay.

### Statistical methods

The primary outcome was specified to be changes in insulin sensitivity (M/I). Key secondary outcomes were changes in endogenous glucose production (%EGP suppression) and changes in lipolysis (% Ra glycerol suppression). Analyses were performed using Graph Pad Prism 7.0. Student’s paired t-test was used to compare baseline values to values after one month of treatment, using two tailed p-values. Values are represented as mean ± SD.

**ClinicalTrials.gov identifier:** NCT02023918

### Changes from ClinicalTrials.gov protocol

The following deviations from the protocol were made. A total of 6 male participants were recruited, enrolled, and completed the entire study. There was a modification to the protocol after the first 4 participants to change the insulin infusion rate during the clamp. This was done because there was near complete suppression of endogenous glucose production with the original insulin dose. However, there did not appear to be a significant effect of lowering the insulin dose in the final 2 participants. To maintain consistency, the final analysis included only the data from the first 4 participants who were treated according to the original clamp protocol. The summary data published on the ClinicalTrials.gov website include all 6 participants, while the data presented here include on the first 4. Despite enrolling fewer than the expected number of participants, the investigators felt that there was an extremely low likelihood that additional participants would change the outcomes, so the study was terminated at this point.

Consort checklist and flowchart are available as Supplementary File 2 and Supplementary File 3.

## Results

### Participants and clinical course

The mean age of the participants was 54.5 ± 2.1 years ( Table 1). Three of the participants carried a diagnosis of hyperlipidemia and were on statin medications. Three of the participants carried a diagnosis of hypertension and two were on antihypertensive medications (amlodipine and metoprolol). One participant carried a diagnosis of gout, but did not require medication during the study.

**Table 1.** Effect of pegvisomant on glucose, lipid, energy metabolism and body composition.

	Baseline	Treatment	*P* value
Characteristics
Age	54.5 ± 2.1		-
Weight (kg)	101.7 ± 5.56	102.5 ± 5.62	0.29
BMI (kg/m^2)	31.2 ± 3.3	31.2 ± 3.21	1.00
IGF-1 (ng/mL)	134.0 ± 41.5	72.0 ± 11.7	**0.04**
IGFBP-3 (mg/L)	1.81 ± 0.30	1.68 ± 0.51	0.71
Energy expenditure
Resting energy expenditure (kcal/day)	2130 ± 93.2	2207 ± 219	0.72
RQ fasting	0.85 ± 0.02	0.81 ± 0.04	**0.04**
RQ clamped	0.85 ± 0.04	0.87 ± 0.03	0.19
Serum lipids
Total cholesterol	160.3 ± 33.3	149.8 ± 20.7	0.42
TG (mg/dL)	199.8 ± 99.7	184.8 ± 70.3	0.46
HDL-cholesterol (mg/dL)	33.5 ± 2.9	31.0 ± 2.5	0.25
LDL-cholesterol (mg/dL)	86.8 ± 35.7	81.8 ± 23.5	0.60
DXA scan
Lean body mass (kg)	67.3 ± 2.3	68.5 ± 1.65	0.17
Total body fat (kg)	30.7 ± 4.2	30.0 ± 4.0	0.12
Truncal fat (kg)	17.6 ± 2.9	17.2 ± 2.4	0.38
Appendicular fat (kg)	11.8 ± 1.3	11.4 ± 1.6	**<0.01**
VAT (g)	1058 ±244	1071 ± 278	0.68
Glucose metabolism
Fasting glucose (mg/dL)	93.5 ± 6.2	94.3 ± 8.7	0.74
Fasting insulin (μIU/mL)	15.5 ± 5.6	16.9 ± 11.5	0.73
HOMA-IR	3.64 ± 1.47	4.10 ± 3.18	0.67
M/I	3.2 ± 1.3	3.4 ± 2.4	0.82
Endogenous glucose production
Fasting Ra glucose (mg/kg • min)	1.83 ± 0.16	2.00 ± 0.25	0.18
Hyperinsulinemic Ra glucose (mg/kg • min)	0.20 ± 0.50	0.36 ± 0.46	**0.01**
Ra glucose suppression by insulin (%)	89.7 ± 26.9	83.5 ± 21.6	0.10
Lipolysis
Fasting Ra glycerol (mg/kg • min)	0.19 ± 0.07	0.20 ± 0.01	0.65
Hyperinsulinemic Ra glycerol (mg/kg • min)	0.07 ± 0.03	0.08 ± 0.03	0.32
Ra glycerol suppression by insulin (%)	59.4 ± 22.1	61.2 ± 14.4	0.67

Data are mean ± SD. *P* values are derived from paired t-tests. Values that are bolded are statistically significant. IGF-1 , insulin like growth factor-1; IGFBP-3, insulin like growth factor binding protein-3; RQ, respiratory quotient; TG, triglycerides; VAT, visceral adipose tissue; M/I, M-value is defined as average glucose infusion rate over a period 80–120 minutes from start of insulin infusion. M/I, ratio M-value to insulin; Ra, rate of appearance.

Participants were adherent to the daily self-infections of pegvisomant based on weekly medication reconciliation and measurement of IGF-1 levels.

### IGF-1 and binding proteins

**Figure 1.**
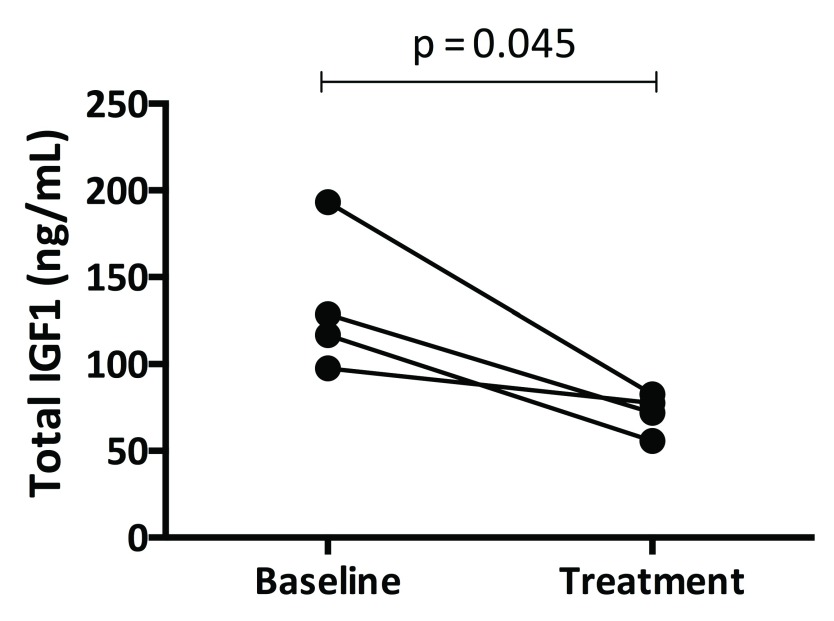
Effects of pegvisomant treatment on total insulin-like growth factor (IGF)-1 levels. IGF-1 decreased as expected over the four-week treatment period. Circles indicate individual baseline and post-treatment values. *P* values are derived from paired t-tests.

As shown in Figure 1, total IGF-1 levels decreased in all participants (134.0 ± 41.5 vs. 72 ± 11.7 ng/mL, p = 0.04). There was no significant change in IGF-BP3 levels ( Table 1).

### Glucose metabolism

**Figure 2.**
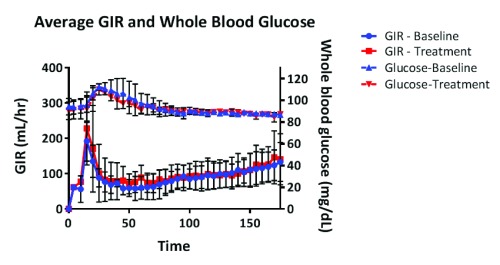
Glucose infusion rate and plasma glucose during hyperinsulinemic euglycemic clamp. There was no difference between the glucose infusion rate (GIR) after treatment with pegvisomant. Blue symbols indicate GIR at baseline. Red symbols indicate GIR post-treatment.

**Figure 3.**
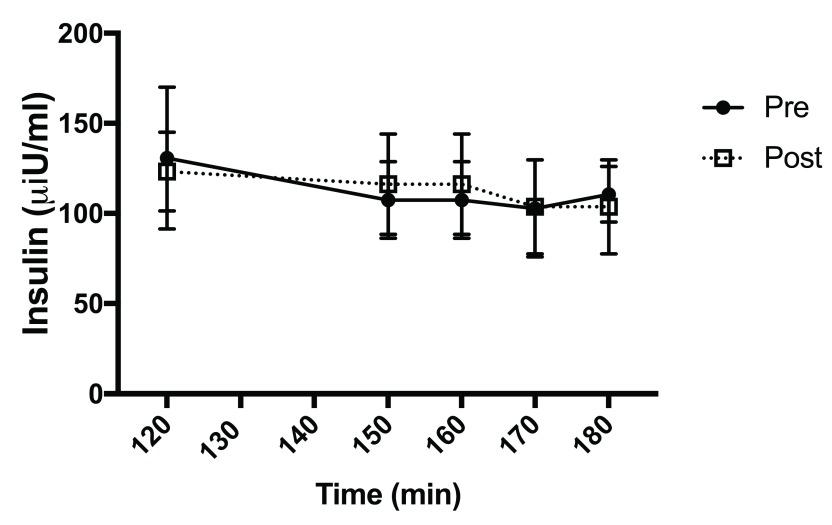
Clamped insulin values. Insulin was measured at various times during the clamp. Data represent the mean insulin levels +/- SD for the pre- (closed circles) and post- (dark squares) during the steady state portion of the clamp.

**Figure 4.**
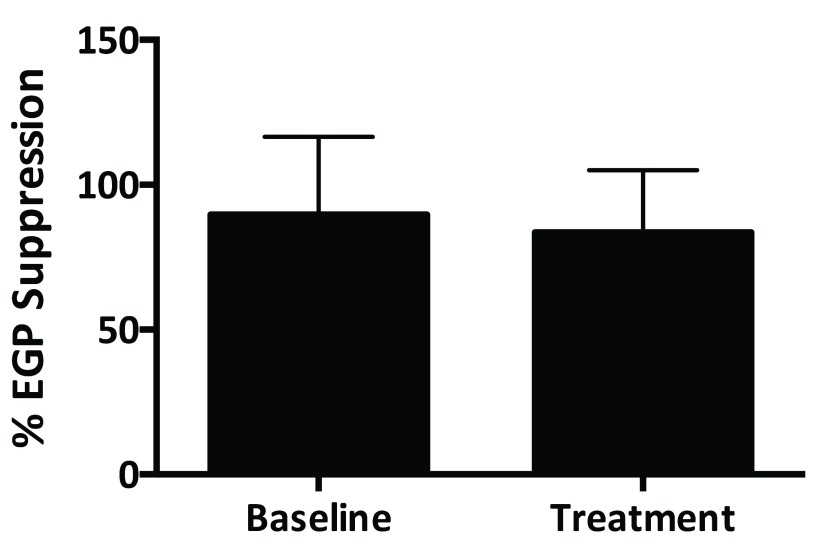
Endogenous glucose production (EGP) suppression. Data represent the percent suppression of endogenous glucose production during the hyperinsulinemic euglycemic clamp at 140 minutes at baseline and after treatment with pegvisomant for one month.

There was no significant change in fasting blood glucose, fasting insulin, or HOMA-IR following four weeks of pegvisomant treatment ( Table 1). There was no significant difference in the serum glucose level or the glucose infusion rate during the clamp ( Figure 2). There was no difference in clamped insulin levels pre- and post-treatment ( Figure 3). There was no difference in basal EGP pre- or post-pegvisomant treatment or in the percent suppression of EGP by insulin ( Figure 4). There was a small increase in Ra glucose post-treatment, but there was no significant difference in suppression of EGP during the clamp ( Table 1). There was no significant change in whole body insulin sensitivity as assessed by M/I (3.2 ± 1.3 vs. 3.4 ± 2.4 p = 0.82).

### Lipid metabolism

There was no significant difference in fasting TG, HDL, or LDL following pegvisomant treatment. Whole body lipolysis did not change in either the fasting state or during hyperinsulinemia.

### Body composition

Lean body mass did not change significantly during the treatment period. There was no change in total fat mass, nor was there a change in visceral adipose tissue mass, but there was a small but statistically significant decrease in appendicular fat (decrease of 0.4 kg, p <0.01). While truncal fat also decreased by 0.5 kg, this did not reach statistical significance (p = 0.11).

### Indirect calorimetry

There was no significant change in resting energy expenditure or RQ measured during the clamp. Fasting RQ declined significantly (p = 0.04).

### Safety measures

One participant, who had prior aspartate aminotransferase (AST) and abnormal liver function (ALT), had a mild increase of his transaminases during weeks 2 and 3 of monitoring, but these remained less than twice the upper limits of normal and decreased back to his baseline while on drug treatment. Side effects for all participants were limited to injection site discomfort. No participants discontinued the drug as a consequence of side effects or laboratory abnormalities.

De-identified raw metabolic data for the four participants Click here for additional data file. Copyright: © 2017 Lee AP et al. 2017 Data associated with the article are available under the terms of the Creative Commons Zero "No rights reserved" data waiver (CC0 1.0 Public domain dedication).

## Discussion

In 1931, the Argentinian physician scientist, Bernardo Houssay demonstrated that injection of anterior pituitary extract worsened glycemic control in dogs ^22, 23^. He also showed that impaired anterior pituitary function led to hypoglycemia and increased sensitivity to insulin ^24^. Houssay and others showed that hypophysectomy ameliorated not only insulin resistance, but also diabetic complications in humans ^25– 29^. Several decades later, GH was shown to confer much of the pituitary-derived diabetogenic activity ^30^. Both loss- and gain-of-function studies in humans and rodents support a role for GH in the biology of insulin responsiveness. While there are a small number of studies exploring the role of GH signaling on insulin resistance in patients with acromegaly, there are no published studies examining the effects of GH antagonism in insulin resistant, non-acromegalic patients. Therefore, we aimed to determine how antagonism of GH signaling with pegvisomant would affect insulin sensitivity in insulin resistant, but non-diabetic men. We found that one month of treatment with the potent GHR antagonist, pegvisomant, reduced levels of circulating IGF-1, but had no effect on insulin sensitivity, endogenous glucose production or lipolysis.

Interestingly, there was a small but statistically significant decrease in appendicular fat mass post-pegvisomant treatment. In acromegalic patients, both surgical treatment and pegvisomant are known to *increase* adiposity, therefore this decrease was unexpected ^31^. We have no experimental data to account for this result, but one potential explanation would be specific targeting of pegvisomant action to the liver. Pegvisomant treatment is known to increase GH levels due to the suppression of IGF-1 ^32^. Circulating IGF-1 is derived almost exclusively from the liver ^33^. If pegvisomant preferentially blocked GH action in the liver, the compensatory increase in circulating GH would cause unopposed GH action in adipose tissue leading to a paradoxical increase in lipolysis and decreased fat mass. We did not observe a significant change in lipolysis, but declines in both appendicular fat mass and resting RQ are consistent with increased lipolysis, so this possibility remains.

Given the very strong rationale supporting the notion that GHR antagonism would improve insulin sensitivity, we were surprised to find no effect of pegvisomant on insulin sensitivity with the clamp. There are several potential explanations for these results. It is possible the dose of pegvisomant was too low or the duration of treatment too short. We had a small sample size and thus we could have insufficient power to detect a difference, though there was absolutely no difference between pre- and post-treated participants and if anything a worsening of hepatic insulin sensitivity. As discussed above, it is possible that pegvisomant has preferential effects on the liver and has relatively little effect on blocking GH signaling in adipose tissue. We saw no effect on whole-body lipolysis and as noted, observed paradoxical changes in body composition. While there is not yet an answer as to the cell or tissue type that mediates the effect of GH on whole body insulin sensitivity, there is evidence suggesting that the predominant site of action is adipose tissue in both mice ^34^ and humans ^35^. Finally, EGP during hyperinsulinemia was nearly fully suppressed at pre-treatment baseline, which means that we would have a hard time detecting further suppression of EGP. This makes the interpretation of the data more difficult as we expected an improvement in suppression of EGP with pegvisomant treatment.

Our study has several limitations. There were a small number of participants, which potentially amplifies the effect of variable diets, activity or other behaviors. It is notable that the other published studies of pegvisomant using the hyperinsulinemic euglycemic clamp technique were small and yet revealed significant effects ^8, 9, 15^. It is possible our subjects were not sufficiently insulin resistant for us to see an effect of pegvisomant. Finally, as previously discussed, near total suppression of EGP at baseline could have obscured an effect of pegvisomant on improvement of hepatic insulin sensitivity.

This is the first report of GH antagonism in insulin resistant, non-acromegalic human participants. Using gold-standard methodology, we observed no effect on insulin sensitivity. Given the abundance of information from human and animal studies that support a role of GH signaling on insulin and glucose metabolism these results are surprising. However, these results suggest that there is still much to be learned about GH and IGF-1 and effects on metabolism. Future studies will be necessary to further explore these effect(s). In particular, studies in more insulin resistant individuals, such as drug naïve, newly diagnosed patients with T2DM, may be more informative.

## Data availability

The data referenced by this article are under copyright with the following copyright statement: Copyright: © 2017 Lee AP et al.

Data associated with the article are available under the terms of the Creative Commons Zero "No rights reserved" data waiver (CC0 1.0 Public domain dedication). 

Dataset 1: De-identified raw metabolic data for the four participants. doi, 10.5256/f1000research.11359.d159415 ^36^

## Supplementary material

Supplementary File 1: Full trial protocol.

Supplementary File 2: CONSORT checklist.

Supplementary File 3: CONSORT flowchart.
