# Supplementary material for: Growth hormone receptor antagonism with pegvisomant in insulin resistant non-diabetic men: A phase II pilot study
Source: F1000Res. 2017 May 3;6:614. [Version 1] doi: 10.12688/f1000research.11359.1 (PMC5499778; doi:10.12688/f1000research.11359.1)
Supplement: Supplementary file 2 [file f1000research-6-12263-s0001.tgz › fcfdd98a-ec1e-46fe-8295-10783cdce550.pdf]

Study Application (Version 1.8)

1.0 General Information

* Enter the full title of your study:

Role of Growth Hormone Receptor Antagonism in Modulating Insulin Sensitivity in Subjects with Insulin Resistance but Without Diabetes

* Enter the study number or study alias

Peg Insulin Resistance

* This field allows you to enter an abbreviated version of the Study Title to quickly identify this study.

2.0 Add Department(s)

2.1 List departments and/or research programs associated with this study

Primary Dept?	Department Name
(selected)	UCSF - 138310 - M_MED-CARD-CORE
(not selected)	UCSF - 138349 - M_MED-ENDO-SFGH

3.0 Assign key study personnel(KSP) access to the study

3.1 * Please add a Principal Investigator for the study:

Ethan J Weiss

Select if applicable

☐ Department Chair ☐ Resident ☐ Fellow

If the Principal Investigator is a Student, Resident, or Fellow, the name of the Faculty Advisor must be supplied below.

3.2 If applicable, please select the Research Staff personnel:

A) Additional Investigators

Ada P Lee

Study Clinician

Kathleen Mulligan, PhD

Co-Principal Investigator

Elizabeth Murphy

Other Investigator

Morris Schambelan

Co-Principal Investigator

B) Research Support Staff

3.3 Please add a Study Contact:

Ada P Lee

Kathleen Mulligan, PhD

Ethan J Weiss

The Study Contact(s) will receive all important system notifications along with the Principal Investigator. (e.g. The project contact(s) are typically either the Study Coordinator or the Principal Investigator themselves).

3.4 If applicable, please add a Faculty Advisor/Mentor:

3.5 If applicable, please select the Designated Department Approval(s):

Shaun Coughlin Ph.D

Department Chair

Add the name of the individual authorized to approve and sign off on this protocol from your Department (e.g. the Department Chair or Dean).

4.0 Qualifications of Key Study Personnel

4.1 November, 2015 - NEW Definition of Key Study Personnel and CITI Training Requirements: UCSF Key Study Personnel include the Principal Investigator, other investigators and research personnel who are directly involved in conducting research with study participants or who are directly involved in using study participants' identifiable private information during the course of the research. Key Personnel also include faculty mentors/advisors who provide direct oversight to Postdoctoral Fellows, Residents and Clinical Fellows serving as PI on the IRB application.   The IRB requires that all Key Study Personnel complete Human Subjects Protection Training through CITI prior to approval of a new study, or a modification in which KSP are being added. More information on the CITI training requirement can be found on our website.   List the study responsibilities and qualifications of any individuals who qualify as Key Study Personnel (KSP) at UCSF and affiliated sites ONLY by clicking the "Add a new row" button. This information is required and your application will be considered incomplete without it.

KSP Name	Description of Study Responsibilities	Qualifications
Weiss, Ethan J	As PI, Dr. Weiss has primary responsibility over the design and oversight of the study. As such, he will supervise the experiments and be responsible for the overall scientific environment. In addition, he will supervise the management of IRB and other regulatory issues, recruitment, screening, inpatient admissions, organization of databases, and data analysis. He will work with Dr. Schambelan in medical supervision and care of subjects. He will also work to continue the bidirectional nature of the translation incorporating work from the basic science lab with the clinical and back to basic science. He will work with the co-investigators to write and adjust protocols and will organize writing and publication of manuscripts and abstracts.	15 years experience at UCSF designing both basic and clinical research studies. Active physician practicing cardiology in inpatient and outpatient setting with focus on cardio-metabolism.
Mulligan, Kathleen, PhD		More than two decades' experience conducting clinical research at UCSF, including inpatient metabolic ward studies such as the current study. Director of Body Composition, Exercise, Metabolism Core for the UCSF-CTSI Clinical Research Service.

	Dr. Mulligan’s primary area of responsibility in this study is in carrying out the in vivo metabolic assessments in the CRC at SFGH. Together with Dr. Weiss, she will be responsible for overseeing and coordinating all aspects of clinical data collection, including management of IRB and other regulatory issues, recruitment, screening, inpatient admissions, organization of databases, and data analysis. She will work with the study coordinator to develop all study documents including participant files, MD orders, flowsheets, case report forms, sample tracking forms, and computer databases; as well as coordination with the CRC staff to schedule screening and inpatient admissions. During inpatient admissions, she will be responsible for overall monitoring of the daily research procedures, including scientific supervision of the preparations for and performance of the clamp and stable isotope studies. Together with Dr. Weiss, she will coordinate with Dr. Schambelan regarding issues of patient management and safety. Together with Dr. Weiss and all co-investigators, she will participate in all aspects of data analysis and interpretation and preparation of presentations and publications. Dr. Mulligan has been performing clinical studies since 1990, including inpatient metabolic studies in persons with diabetes and insulin resistance.	
Schambelan, Morris		Professor Emeritus of Medicine with decades of experience performing clinical studies; former medical director of the clinical research center at SFGH and, subsequently, for the UCSF-CTSI Clinical Research Centers.

	Dr. Schambelan will be the primary physician responsible for the recruitment of study subjects and for the medical supervision and care of the subjects during their inpatient admissions as well as for safety monitoring during their outpatient treatment. He will specifically advise on medical issues pertaining to subject eligibility, safety monitoring, and diabetes management, which are critical in this population. Dr, Schambelan has an extensive experience in designing and conducting proof-of-principle studies such as described herein and, of particular relevance to this application, in the use of GH and IGF-1 in the treatment of metabolic disorders, Dr. Schambelan is Professor Emeritus of Medicine on active recall status and thus has maintained a continued and robust role as an investigator and physician at SFGH and UCSF. Dr. Schambelan served as Associate Chair for Clinical and Translational Research in the Department of Medicine, Chief of Endocrinology and Metabolism at SFGH, as well as Program Director for the SFGH CRC and, more recently, as Program Director for the multisite CTSI-CRC at UCSF. He has decades of experience in performing intensive metabolic studies such as those in the current project. In addition to his key role as medical supervisor, he will also advise on all other aspects of the study and will actively participate in organization of writing and publication of manuscripts.	
Dr. Murphy, Elizabeth MD	Dr. Murphy has an extensive background in the performance of stable isotope analyses using GC/MS as described in the study protocol. She will be responsible for the supervision of the measurement of these analytes in the Stable Isotope Core of the Division of Endocrinology at SFGH. She is also the current Chief of the Division of Endocrinology and Metabolism at SFGH and, as such, will facilitate access to the research subjects who will be recruited under the direction of Dr. Schambelan.	Dr. Murphy is the Chief of Endocrinology at SFGH where she is an active clinician and cares for patients with diabetes. She is also an experienced clinical and basic researcher with tremendous experience in diabetes and metabolic disorders.
Lee, Ada P		Dr. Lee is a Clinical Endocrinology Fellow who has completed her clinical fellowship and is now in her research years. She has taken care of patients with diabetes and acromegaly and other pituitary disorders.

	Dr. Lee will be a physician responsible for the recruitment of study subjects and for the medical supervision and care of the subjects during their inpatient admissions as well as for safety monitoring during their outpatient treatment. She will specifically advise on medical issues<br>pertaining to subject eligibility, safety monitoring, and diabetes management, which are critical in this<br>population. Dr, Schambelan has an extensive experience in designing and conducting proof-of-principle studies such as described herein and, of particular relevance to this application, in the use of GH and IGF-1 in<br>the treatment of metabolic disorders, Dr. Lee is a Clinical Endocrinology Fellow who has completed her clinical fellowship and is now in her research years.	
Nordstrom, Sarah M	Dr. Nordstrom is a Ph.D. pharmacologist who is currently a senior post-doctoral fellow in the Weiss lab. She will serve as a part-time study coordinator and will be primarily responsible for the orgnanization of the study including screening and eligibility assessments, subject contact, appointment scheduling, follow up and data collection and management.	Dr. Nordstrom is an extremely accomplished pharmacologist who has extensive experience coordinating research.

5.0 Initial Screening Questions - Updated 9/13 (Note: You must answer every question on this page to proceed). If you are converting to the new form, check questions 5.4, 5.6, 5.7, 5.8 and 5.10 before saving and continuing to the next section.

5.1 * Application type:

- (•) Full Committee
- ( ) Expedited
- ( ) Exempt

5.2 * Risk level (Help Text updated 9/13):

- ( ) Minimal risk
- (•) Greater than minimal risk

5.3 * Subject contact:

- (•) Yes (including phone, email or web contact)
- ( ) No (limited to medical records review, biological specimen analysis, and/or data analysis)

5.4 * Funding (past or present):

- (•) Funded or will be funded (external sponsor, gift, program or specific internal or departmental funds)
- ( ) Unfunded (no specific funds earmarked for this project)
- ( ) Unfunded student project

5.5 * The Principal Investigator and/or one or more of the key study personnel has financial interests related to this study:

◯Yes ◉No

If **Yes**, the Conflict of Interest Advisory Committee (COIAC) office may contact you for additional information.

5.6 * This is an investigator-initiated study:

◉Yes ◯No

5.7 * This study ONLY involves retrospective records review and/or identifiable biospecimen analysis:

◯Yes ◉No

5.8 * This is a clinical trial:

◉Yes ◯No

Clinical Trial Registration

"NCT" number for this trial:

NCT02023918

5.9 * This is a multicenter study:

◯Yes ◉No

5.10 * This application involves the study of unapproved or approved drugs, devices, biologics or in vitro diagnostics:

◉Yes ◯No

5.11 * This application involves a Humanitarian Use Device:

◉No
◯Yes, and it includes a research component
◯Yes, and it involves clinical care ONLY

5.12 * This study involves human stem cells (including iPS cells and adult stem cells), gametes or embryos:

◉No
◯Yes, and requires CHR and GESCR review
◯Yes, and requires GESCR review, but NOT CHR review

5.13 * This is a CIRB study (e.g. the NCI CIRB will be the IRB of record):

◯Yes ◉No

5.14 * This application includes a request to rely on another IRB (other than NCI CIRB):

◯Yes ◉No

Note: If this request is approved, the CHR will **NOT** review and approve this study. Another institution will be the IRB of record.

6.0 Funding

6.1 Identify all sponsors and provide the funding details. If funding comes from a Subcontract, please list only the Prime Sponsor: Note: we require only a P Number OR an A Number for funding coming through UCSF. Please avoid these common errors in funding documentation: DO NOT add the A Number if a P Number was already provided OR update the A Number field when a new funding cycle begins. The IRB does NOT use this information or want these changes made. DO NOT add a grant continuation as a new funding source.

External Sponsor:

	Sponsor Name	Sponsor Type	Funding Through	Contract Type:	UCSF RAS "P number" or eProposal number	UCSF RAS System Award Number ("A" + 6 digits)
—	Pfizer	04	UCSF	Contract	P0000000	

Sponsor Name:	Pfizer
Sponsor Type:	04
Sponsor Role	Funding
Grant/Contract Number	
Awardee Institution	UCSF
Is Institution the Primary Grant Holder:	Yes
Contract Type:	Contract
UCSF RAS "P number" or eProposal number	P0000000
UCSF RAS System Award Number ("A" + 6 digits)	
Grant Number for Studies Not Funded thru the institution:	
Grant Title:	Role of Growth Hormone Receptor Antagonism in Modulating Insulin Sensitivity in Subjects with Insulin Resistance but Without Diabetes
Award Recipient:<br>If Award Recipient is not the same as identified on the study.	
Explain Any Significant Discrepancy:	

Gift, Program, or Internal Funding (check all that apply):

☐ Funded by gift (specify source below)
☐ Funded by UCSF or UC-wide program (specify source below)
☐ Specific departmental funding (specify source below, if applicable)

List the gift, program, or departmental funding source:

6.2 If you tried to add a sponsor in the question above and it was not in the list, follow these steps: If funding has already been awarded or the contract is being processed by the Office of Sponsored Research (OSR) or Industry Contracts Division (ICD), your sponsor is already in the system and the project has an eProposal Proposal or Award number. Check with your department's OSR Staff or ICD Officer to ask how the sponsor is listed in the UC sponsor list and what the Proposal or Award number is. Click here to find your OSR staff and here to find your ICD staff. If your sponsor is not yet in the list, enter it in the box below.

○ Sponsor not in list

Only if your sponsor is not yet in the list, type the sponsor's name:

If the funding is administered by the UCSF Office of Sponsored Research, your study will not receive CHR approval until the sponsor and funding details have been added to your application.

6.3 * This study is currently supported in whole or in part by Federal funding OR has received ANY Federal funding in the past (Help Text updated 9/13):

○ Yes ◉ No

If **yes**, indicate which portion of your grant you will be attaching:

☐ The Research Plan, including the Human Subjects Section of your NIH grant or subcontract
☐ For other federal proposals (contracts or grants), the section of the proposal describing human subjects work

☐ The section of your progress report if it provides the most current information about your human subjects work
☐ The grant is not attached. The study is funded by an award that does not describe specific plans for human subjects, such as career development awards (K awards), cooperative agreements, program projects, and training grants (T32 awards) OR UCSF (or the affiliate institution) is not the prime recipient of the award

7.0 Sites

7.1 Institutions (check all that apply):

☑ UCSF
☐ China Basin
☐ Helen Diller Family Comprehensive Cancer Center
☑ Mission Bay
☐ Mount Zion
☑ San Francisco General Hospital (SFGH)
☐ SF VA Medical Center (SF VAMC)
☐ Blood Centers of the Pacific (BCP)
☐ Blood Systems Research Institute (BSRI)
☐ Fresno (Community Medical Center)
☐ Gallo
☐ Gladstone
☐ Institute on Aging (IOA)
☐ Jewish Home
☐ SF Dept of Public Health (DPH)

7.2 Check all the other types of sites not affiliated with UCSF with which you are cooperating or collaborating on this project (Help Text updated 9/13):

☐ Other UC Campus
☐ Other institution
☐ Other community-based site
☐ Foreign Country

List the foreign country/ies:

7.3 Check any research programs this study is associated with:

☐ Cancer Center
☐ Center for AIDS Prevention Sciences (CAPS)
☐ Global Health Sciences
☐ Immune Tolerance Network (ITN)
☐ Neurosciences Clinical Research Unit (NCRU)
☐ Osher Center
☐ Positive Health Program

8.0 Study Design

8.1 * Study design (Help Text updated 9/13):

This is a pilot open label trial of pegvisomant (a growth hormone receptor antagonist) in the treatment of type 2 diabetes. A total of 12 men and women ages 20-65 with insulin resistance who are not currently on pharmacologic treatment will be recruited to the study. Prior to treatment, subjects will be hospitalized in the Clinical and Translational Science Institute (CTSI) Clinical Research Center (CRC) at San Francisco General Hospital to undergo comprehensive studies of carbohydrate and lipid metabolism, energy expenditure and

substrate utilization, circulating free fatty acid levels, and the growth hormone axis. At the conclusion of baseline testing, subjects will receive open-label treatment with pegvisomant. After 4 weeks of treatment, subjects will be readmitted to the CRC, and baseline measurements will be repeated .

8.2 If this is a clinical trial, check the applicable phase(s) (Help Text updated 9/13):

- [ ] Phase I
- [x] Phase II
- [ ] Phase III
- [ ] Phase IV

9.0 Scientific Considerations

9.1 Hypothesis (Help Text updated 9/13):

This study has a hypothesis:

(•) Yes ( ) No

If yes, state the hypothesis or hypotheses:

1. **We hypothesize that treatment with pegvisomant will improve hepatic insulin sensitivity in patients with insulin resistance**.
2. **We hypothesize that treatment with pegvisomant will improve extra-hepatic insulin sensitivity as well.**
3. **We hypothesize that treatment with pegvisomant will inhibit basal lipolysis and will decrease circulating free fatty acid levels.**

9.2 * List the specific aims:

Aim 1. What is the effect of GHR antagonism on hepatic insulin sensitivity in patients with insulin resistance?Our preliminary studies in mice suggest that GH signaling modulates hepatic insulin sensitivity as evidenced by changes in insulin-mediated suppression of endogenous glucose production (EGP). To determine the effect of pegvisomant on EGPunder both fasting conditions and during hyperinsulinemia, we will perform stable isotope tracer studies coupled with a euglycemic hyperinsulinemic clamp before and after 4 weeks of treatment with pegmisovant.

Aim 2. What is the effect of pegvisomant on peripheral insulin resistance in patients with insulin resistance? GH has also been shown to impair insulin sensitivity in muscle and adipose tissue. To determine the effect of pegvisomant on insulin-mediated glucose uptake in extra-hepatic tissues, we will perform a euglycemic hyperinsulinemic clamp in patients with insulin resistance as described above.

Aim 3. What is the effect of pegvisomant on lipolysis in patients with insulin resistance? GH stimulates lipolysis and mobilizes free fatty acids (FFA). High levels of FFA in turn inhibit insulin signaling, prompting some to speculate that the diabetogenic effects of GH might relate to effects of increasing levels of FFA. To assess this, we will perform stable isotope studies of whole-body lipolysis before and after 4 weeks of pegvisomant treatment. We will measure levels of plasma FFA in the fasting and clamped states, and we will explore the association of these with changes in hepatic and extra-hepatic insulin sensitivity. We hypothesize that treatment with pegvisomant will inhibit basal lipolysis.

9.3 Statistical analysis:

We have developed our statistical plan and sample size estimates with consulation and assistance by Dr. Joel Simon of the UCSF CTSI. We will continue to work closely with CTSI through the course of the study. Each subject will serve as his/her own control.

Statistical Analyses: Primary interest will focus on changes in fasting and dynamic measures of glucose and lipid metabolism from baseline to week 4 by calculation of mean changes, 95% confidence intervals, and p-values from the paired t-test. The primary outcome will be endogenous glucose production (EGP). If parametric assumptions for these methods are not met, then median changes with 95% confidence intervals will be calculated, and p-values will be obtained from the Wilcoxon signed-rank test.

In addition, multivariate models of changes that control for baseline values and on-study measurements other possible predictors will be examined. For example, changes in EGP will be modeled by multivariate linear regression, with particular attention given to change in FFA levels as a potential explanatory covariate . If estimated treatment effects are reduced or eliminated when controlled for such covariates, this may suggest a mechanism. Multivariate models will be limited to two or at most three predictors in a model to guard against over-fitting the limited amount of data.

Although many measurements will be examined and many comparisons made, no formal multiple comparisons adjustments will be made. The measures and comparisons that we propose are all closely related and focused on specific questions, facts that would be ignored by formal adjustments such as the Bonferroni method. Instead, over-interpretation of isolated findings of statistical significance will be avoided by examining the directions and magnitudes of all the estimated effect and by interpreting them in light of the biological relationships among them.

Sample Size: For the primary outcome (EGP), enrollment of 12 subjects will provide 80% power by a paired t-test to detect a standardized effect size (mean change from baseline divided by the standard deviation of the change) of 0.9. Because the metabolic assessments are highly sensitive and are performed under carefully controlled conditions, and our focus on changes within each subject automatically controls for inherent differences between subjects, we expect that variability in the within-subject changes will be small enough to allow detection of significant changes using the relatively small sample size appropriate for this proof-of-principle study. We have successfully employed this study design in other proof-of-principle studies testing novel pharmacologic treatments. For example, significant changes in insulin sensitivity were seen in one study from our laboratory, in which GH was administered to *six* HIV-positive patients with fat accumulation. Data from that study yielded standardized effect sizes of 1.11 (M/I), 1.15 (EGP), and 2.59 (lipolysis) for the changes during treatment. Because all of these values exceed the standardized effect size of 0.9 that we can detect within a group of 12, we expect to have greater than 80% power to detect significant changes in all of these outcomes.

9.4 If this study has undergone scientific or scholarly review, please indicate which entity performed the review:

☐ Cancer Center Protocol Review Committee (PRC) (Full approval is required prior to final CHR approval for cancer-related protocols.)
☑ CTSI Clinical Research Center (CRC) advisory committee
☑ Departmental scientific review
☐ Other:

Specify
Other
:

10.0 Background

10.1 Background:

<u>Hepatic Insulin Resistance is an Important Risk Factor for Diabetes</u>

The worldwide incidence of type 2 diabetes (T2DM) continues to increase dramatically [11]. Insulin resistance plays a critical role in the pathogenesis of T2DM, but the mechanism(s) underlying insulin resistance in target tissues remain complex and unresolved [12] Insulin regulates the metabolism of glucose, lipids and proteins in multiple target tissues including liver, muscle and fat [13]. In the liver, insulin acts directly on

hepatocytes to inhibit gluconeogenesis and to stimulate glycogen synthesis (hepatic glucose production [HGP]) and to stimulate fatty acid synthesis. Hepatic insulin resistance is an important risk factor for the development of T2DM and has also been associated with nonalcoholic fatty liver disease, a known risk factor for cardiovascular disease [8,14]. Mice with liver-specific deletion of the insulin receptor develop dramatic insulin resistance and an impairment in the ability of insulin to suppress HGP [15]. Furthermore, hepatic insulin resistance leads to dyslipidemia and increased risk of atherosclerosis [16,17]. Elevated levels of plasma FFAs can lead to impaired hepatic insulin sensitivity through inhibition of insulin signaling [9,18]. Yet there are few drugs available to augment insulin sensitivity. Indeed, most of these drugs have untoward effects on body composition and/or lipids.

Growth Hormone (GH) Signaling Impairs Insulin Sensitivity and Increases Risk of T2DM

Growth hormone (GH) is a known regulator of lipid and carbohydrate metabolism. GH induces dimerization and activation of the GH receptor (GHR). Activated GHR recruits and activates Janus kinase 2 (JAK2) which, in turn, activates sinnal transducer and activator of transcription factor family members (STAT5) to regulate transcription of target genes including IGF-1 [19]. In mice, global deletion of the GHR leads to improved insulin sensitivity, lower fasting glucose and insulin levels, and increased longevity despite an increase in body fat [2]. Mice with global disruption of GHR are protected from high fat diet-induced changes in carbohydrate metabolism despite normal acquisition of body fat [2]. Humans with inactivating mutations in GHR (so-called Laron syndrome) show similar changes in carbohydrate metabolism with improved insulin sensitivity and apparent protection from T2DM despite increased body fat [3]. The direct metabolic consequences of GH signaling remain complicated and *in vivo* studies are difficult to interpret because of the multiple feedback/feed forward loops. What is known is that GH excess causes insulin resistance [20] and GH replacement in growth hormone deficient (GHD) patients leads to impaired glucose tolerance and an increased risk of diabetes [10,21-23]. GH itself may also increase HGP [24,25]. The mechanisms of GH-mediated insulin resistance remain unclear though most agree that a predominant and important site of insulin resistance is the liver [26].

GH Can Antagonize Insulin Sensitivity Directly in Peripheral Tissues

GH is well-known to antagonize insulin signaling in cells [20]. The insulin receptor (IR) is a protein tyrosine kinase that is autophosphorylated after ligand binding, leading to phosphorylation of a host of intracellular proteins including insulin receptor substrate (IRS)-1 and IRS-2 [27]. GH has been shown to mediate skeletal [28] and adipose [29] tissue insulin resistance via upregulation of the phosphoinositide 3-kinase regulatory subunit p85 (*Pik3r1*). *Pik3r1*is also one of the most highly GH-regulated genes in adipocytes and is an integral component of the insulin signaling pathway, negatively regulating PI3K activity [28,30]. We have also learned a great deal about the crosstalk between GH and insulin signaling from mouse models. As described above, mice with global deletion of GHR have increased insulin sensitivity despite relative obesity [2,31]. Recent work from other groups has shown conflicting results regarding the effect of specific deletion of GHR in skeletal muscle on whole body insulin sensitivity [32,33]. Several groups (including ours) have knocked out the GHR or necessary downstream signaling components in hepatocytes [34-38]. While each group has found that liver-specific disruption of GH signaling unequivocally leads to profound hepatic steatosis, the net effects on carbohydrate metabolism have been mixed. The interpretation of such findings is extremely difficult given the constellation of changes that occur in mice with hepatic inactivation of GH signaling. Notably, there is a dramatic increase in circulating GH levels with concomitant effects on peripheral tissues and on lipolysis, and there is a profound increase in liver lipid content. Because of the dramatic effects of GH on fasting insulin levels, there has been great interest on the potential direct effect of GH signaling on insulin synthesis or secretion from pancreatic beta cells. Mice with beta-cell specific disruption of GHR were recently described, and on normal chow, there is little effect on fasting insulin levels or insulin content in the pancreas [39]. There are no previously published reports of mice with adipocyte-specific alterations in GH signaling.

GH Stimulates Lipolysis; Free Fatty Acids Promote Insulin Resistance

GH mobilizes energy from adipocytes in the form of plasma free fatty acids (FFA) [40]. When administered to people or animals, GH reduces fat mass, presumably by increasing the rate of lipolysis [10,25,41-44]. The precise mechanism whereby GH regulates lipolysis remains unknown. Mice or people with GH deficiency or with abnormal signaling through GHR have increased body fat. Although the potent lipolytic effects of GH have been well-recognized, the exact role(s) of GH signaling in lipid flux in health and disease is not completely understood. One particularly attractive theory is that GH modulates insulin resistance in target tissues through mobilization of FFA. The so-called Randle hypothesis was first proposed in 1963 [45]. Since

then, much attention has been focused on the theory that increased levels of FFA can promote insulin resistance. It has been shown that high levels of FFA inhibit insulin's ability to suppress HGP [9]. The precise mechanism underlying this observation remains unknown, but it is thought to occur through effects on IRS-1 [46]. Further, this phenomenon underlies the widely-held belief that the diabetogenic effects of GH are consequence of increasing levels of FFA [9,10].

Pegvisomant Improves Insulin Sensitivity in Patients with Acromegaly

Acromegaly is a rare but devastating disease resulting from increased GH secretion. Decreasing GH levels with surgery or medical therapy improves morbidity and mortality [6,47]. Patients with acromegaly have impaired glucose tolerance associated with both hepatic and peripheral insulin resistance and an increased risk of T2DM [4-6]. Pegvisomant is a novel specific GHR antagonist that is currently approved for the medical treatment of acromegaly [48,49]. Pegvisomant normalizes IGF1 levels in the majority of patients with acromegaly [50], and this in turn leads to improvements in insulin and glucose metabolism [51] despite significantly increased GH levels. Pegvisomant improves both hepatic and peripheral insulin resistance in acromegalic patients [52,53]. While pegvisomant has also been studied in healthy volunteers, it has not been studied in patients with T2DM who do not have acromegaly. Studies in healthy subjects did not reveal any significant changes in markers of fasting insulin or glucose homeostasis [54,55]. However, hyperinsulinemic euglycemic clamp studies have not been performed in healthy volunteers taking pegvisomant and there have been no studies looking at the effects on lipolysis in normal or acromegalic subjects.

Significance

GH has powerful effects on carbohydrate and lipid metabolism. Because of its favorable effects on body composition, GH has been used to "treat" abdominal obesity and other conditions [56-59]. However, GH administration leads to insulin resistance and an increased risk of diabetes. Conversely, antagonism of GH signaling using the specific antagonist, pegvisomant, improves carbohydrate metabolism in patients with acromegaly. Therefore, it is clear that excess GH or GH signaling impairs insulin sensitivity and increases the risk for diabetes, while blocking GH signaling can improve carbohydrate metabolism. However, human and animal data also suggest that basal GH signaling serves as a potent negative regulator of insulin sensitivity. In mice, deletion of GHR protects mice from insulin resistance due to high fat diet [2]. In preliminary work summarized below, we have shown that this occurs due to changes in GH signaling in adipose tissue. This suggests that antagonism of GH signaling might improve insulin sensitivity in patients without acromegaly. To date, there are no published studies in which pegvisomant or other specific GHR antagonists have been given to patients with insulin resistance or T2DM but without acromegaly. With the exploding epidemics of obesity and T2DM, understanding novel mediators of insulin resistance is extremely important. Furthermore, discovery of new approaches to improve insulin sensitivity in patients with or at risk for T2DM remains a vitally important goal. The pilot study proposed here should provide important insights into the role of the GH/IGF1 pathway in modulating carbohydrate metabolism in T2DM. This should afford both improved knowledge of the pathophysiology and may ultimately lead to novel approaches aimed at the treatment of insulin resistance and diabetes.

10.2 Preliminary studies:

Figures for this section will be included as an attachment

Mice with global deletion of GHR and people with inactivating mutations in GHR both display improved insulin sensitivity and protection from T2DM. Conversely, mice or people with increased GH levels or increased GH signaling are insulin resistant. How and where GH signaling modulates insulin sensitivity *in vivo* remains unknown. To begin to unravel the mechanism(s) underlying the effects of GH signaling on insulin signaling, we and others have made a series of mouse models with tissue-specific inactivation of GH signaling.

<u>Disrupting Hepatic GH Signaling Causes Hepatic Insulin Resistance</u>

As reported by us and others, mice with liver-specific disruption of GH signaling have a vast array of abnormalities in insulin/glucose metabolism [34,35,38]. Our own model employs the hepatocyte-specific disruption of JAK2 (so-called JAK2L) [35]. In preliminary work, we have observed that JAK2L mice have increased fasting plasma insulin (**Fig. 1a**) and glucose (**Fig. 1b**) levels, display normal glucose tolerance (**Fig. 1c**), but have profound whole-body insulin resistance (**Fig 1d**) in comparison to control (CON) mice.

Utilizing aeuglycemic-hyperinsulinemic clamp, we found that there was a dramatic decrease in the glucose infusion rate (GIR) (**Fig. 2a**), an increase in EGP under both basal (**Fig. 2b**) and hyperinsulinemic conditions (**Fig. 2c**), and a dramatic decrease in the percent suppression of EGP by insulin (**Fig. 2d**) in JAK2L mice versus CON.

In data not shown, we found no significant difference in 2-deoxy glucose uptake in skeletal muscle. Taken together, these results suggest that deletion of JAK2 from hepatocytes leads to hepatic insulin resistance. However, accurate interpretation of the effects of liver-GH signaling on whole-body carbohydrate metabolism is complex [35]. Loss of hepatic GH signaling leads to a near total decrease in circulating IGF1 and a concomitant increase in GH levels. In addition, there is an increase in the rate of lipolysis and an increase in plasma FFA. Finally, the animals all have profound hepatic steatosis.

<u>Concomitant Disruption of GH Signaling in Adipocytes Improves Hepatic Insulin Resistance in JAK2L Mice</u>

To isolate the effects of hepatic GH signaling on the profound hepatic insulin resistance observed in JAK2L mice, we generated mice with adipocyte-specific deletion of JAK2 using an adiponectin *Cre* (JAK2A). In data not shown here (see abstracts 1 and 2), we demonstrated that these mice were unresponsive to GH in adipose tissue. Further, JAK2A mice were resistant to GH-stimulated lipolysis, and the basal rate of lipolysis was decreased as compared to CON. There was a modest increase in percent body fat. Next, we crossed JAK2L and JAK2A mice. Since GH-stimulated lipolysis was reduced in JAK2A mice, we reasoned that JAK2L/A mice would have normal plasma FFA and thus might have normal liver lipid content despite very high GH levels. Indeed, in data not shown here, there was a near-complete reduction in liver TG content in JAK2L/A mice as compared to JAK2L. Furthermore, there were no differences in plasma FFA. IGF1 and GH levels were identical to those seen in JAK2L mice.

We then comparedthe measures of insulin/glucose metabolism in CON and JAK2L/A mice. Again, the notable differences between the JAK2L and JAK2L/A mice were the normalization of liver lipid, the reduction in plasma FFA, and the reduction in the rate of lipolysis, all due to the loss of JAK2 in adipocytes. Similarities in the two models include the decrease in IGF1 and the increase in GH levels, and the concomitant effects of either on peripheral tissues. As seen in **Fig. 3**, there were no significant differences in fasting plasma insulin (**Fig. 3a**) or glucose (**Fig. 3b**) levels, glucose tolerance (**Fig. 3c**), or whole-body insulin tolerance (**Fig 3d**) between CON and JAK2L/A mice.

Utilizing euglycemic-hyperinsulinemic clamps, we found that there were no differences in the GIR (41.4 vs. 41.5 mg/(kg-min), *ns*), EGP under either basal (12.4 vs. 12.5 mg/(kg-min), *ns*) or hyperinsulinemic (4.9 vs. 4.0 mg/(kg-min), *ns*) conditions, or the percent suppression of EGP by insulin (58.7 vs. 66.6%, *ns*) between CON and JAK2L/A mice, respectively. This suggests that the profound difference in insulin sensitivity (hepatic) observed in JAK2L mice was most likely due to either the difference in liver lipid, the difference in plasma FFA, the difference in the rate of lipolysis, or some other factor that was affected by the disruption of

JAK2 in adipocytes. Recall that the levels of plasma GH and IGF1 are not different between JAK2L and JAK2LA mice suggesting that the difference is not likely mediated by effects of GH on peripheral insulin sensitivity. Indeed, in data not shown, there were no differences in 2-deoxyglucose uptake in either skeletal muscle or adipose tissue in JAK2L/A mice versus CON. Finally, since JAK2 is deleted from hepatocytes in both JAK2L and JAK2L/A mice, we can safely say that there is no effect of GH signaling on modulating insulin sensitivity directly in hepatocytes.

Disruption of GH Signaling Specifically in Adipocytes Confers Increased Insulin Sensitivity

The above data show that high levels of GH mediate hepatic insulin resistance. However, the data also suggest that this effect is not mediated by the direct action of GH on hepatocytes. Therefore, the question as to where and how GH signaling modulates insulin sensitivity remains unanswered. To explore the role of GH-stimulated lipolysis on insulin sensitivity, we next compared CON and JAK2A mice. Here, we observed a trend toward decreased fasting insulin (**Fig. 4a**), a modest decrease in fasting glucose (**Fig. 4b**), no difference in glucose tolerance (**Fig. 4c**), and an improvement in whole-body insulin tolerance (**Fig. 4d**). Results from euglycemic-hyperinsulinemic clamp studies showed that there was an increase in GIR (**Fig. 5a**), no difference in basal EGP (**Fig. 5b**), but a dramatic decrease in EGP during hyperinsulinemia (**Fig. 5c**), and an increase in suppression of EGP by insulin (**Fig. 5d**) in JAK2A mice versus CON. These exciting results suggest that disruption of JAK2 in adipocytes confers an improvement in hepatic insulin sensitivity. We have not yet established the underlying mechanism but, on face value, it appears that GH modulates hepatic insulin sensitivity via effects on adipose tissue. This could be due to effects on GH-stimulated lipolysis or another as yet undefined mechanism.

Disruption of JAK2 from Adipocytes Confers Protection from Diet-Induced Insulin Resistance

In the above preliminary data, we found that disruption of GH signaling in hepatocytes leads to significant hepatic insulin resistance in mice fed normal chow. Compound disruption of GH signaling from adipocytes and hepatocytes restored normal hepatic insulin sensitivity. Moreover, disruption of GH signaling in adipose tissue alone led to improved hepatic insulin sensitivity. Combined with the data from GHR global knockout mice as well as in patients with Laron syndrome, it appears that abrogation of GH signaling in fat leads to improved hepatic insulin sensitivity. To determine how disruption of JAK2 from adipocytes might affect insulin sensitivity in a challenged state, we put all 4 groups of animals (CON, JAK2L, JAK2A, JAK2L/A) on high fat diet (HFD) for 10 weeks. There was an increase in percent body fat in all four groups, but the increase in body fat in the JAK2L group was significantly less than the other three (probably due to high GH). However, despite the lack of protection from HFD-induced obesity, both JAK2A and JAK2L/A animals were dramatically protected in terms of whole body insulin sensitivity (**Fig. 6**). This protection occurred despite an increase in skeletal muscle lipid content and did not appear to relate to changes in liver lipid content. In fact, JAK2A mice were dramatically protected from HFD-induced hepatic steatosis (see abstract 2). We eagerly await the results of the clamp studies for the HFD cohort which are currently in progress. However, based on the data already presented, we predict that deletion of JAK2 from adipocytes will confer improved hepatic insulin sensitivity.

In summary, disruption of GH signaling has favorable effects on insulin/glucose homeostasis in mice and humans. To explore the mechanisms underlying these effects, we have used sophisticated genetic models in mice to isolate the specific cells and tissues wherein the loss of GH signaling impacts carbohydrate metabolism. As described above, we have found that disruption of GH signaling in mouse adipose tissue recapitulates much of the benefit seen in mice with global deletion of GHR. Specifically, disruption of GH signaling in fat improves hepatic insulin sensitivity. Furthermore, mice with fat-specific disruption of GH signaling are protected from high-fat diet induced diabetes. These results are powerful and provide a strong rationale to determine the effect of GHR antagonism in people as a means to improve insulin resistance associated with T2DM.

10.3 References:

1. Liu J-L, Coschigano KT, Robertson K, et al. Disruption of growth hormone receptor gene causes diminished pancreatic islet size and increased insulin sensitivity in mice. *Am J Physiol Endocrinol Metab.* September 1, 2004 2004;287(3):E405-413.

2. List EO, Sackmann-Sala L, Berryman DE, et al. Endocrine Parameters and Phenotypes of the Growth Hormone Receptor Gene Disrupted (GHR /) Mouse. *Endocrine Reviews.* June 1, 2011

2011;32(3):356-386.

3. Guevara-Aguirre J, Balasubramanian P, Guevara-Aguirre M, et al. Growth Hormone Receptor Deficiency Is Associated with a Major Reduction in Pro-Aging Signaling, Cancer, and Diabetes in Humans. *Science Translational Medicine.* February 16, 2011 2011;3(70):70ra13.

4. SÖNKSEN PH, GREENWOOD FC, ELLIS JP, LOWY C, RUTHERFORD A, NABARRO JDN. Changes of Carbohydrate Tolerance in Acromegaly with Progress of the Disease and in Response to Treatment. *Journal of Clinical Endocrinology & Metabolism.* October 1, 1967 1967;27(10):1418-1430.

5. Møller N, Schmitz O, Jøorgensen JO, et al. Basal- and insulin-stimulated substrate metabolism in patients with active acromegaly before and after adenomectomy. *Journal of Clinical Endocrinology & Metabolism.* May 1, 1992 1992;74(5):1012-1019.

6. Holdaway IM, Rajasoorya RC, Gamble GD. Factors Influencing Mortality in Acromegaly. *Journal of Clinical Endocrinology & Metabolism.* February 1, 2004 2004;89(2):667-674.

7. Vijayakumar A, Yakar S, Leroith D. The Intricate Role of Growth Hormone in Metabolism. *Frontiers in Endocrinology.* 2011-September-27 2011;2(32):1-11.

8. Samuel VT, Petersen KF, Shulman GI. Lipid-induced insulin resistance: unravelling the mechanism. *The Lancet.* 2010;375(9733):2267-2277.

9. Kovacs P, Stumvoll M. Fatty acids and insulin resistance in muscle and liver. *Best Practice & Research Clinical Endocrinology & Metabolism.* 2005;19(4):625-635.

10. Vijayakumar A, Novosyadlyy R, Wu Y, Yakar S, LeRoith D. Biological effects of growth hormone on carbohydrate and lipid metabolism. *Growth Hormone & IGF Research.* 2010;20(1):1-7.

11. Chen L, Magliano DJ, Zimmet PZ. The worldwide epidemiology of type 2 diabetes mellitus[mdash]present and future perspectives. *Nat Rev Endocrinol.* 2012;8(4):228-236.

12. Samuel Varman T, Shulman Gerald I. Mechanisms for Insulin Resistance: Common Threads and Missing Links. *Cell.* 2012;148(5):852-871.

13. Cheatham B, Kahn CR. Insulin Action and the Insulin Signaling Network. *Endocrine Reviews.* April 1995 1995;16(2):117-142.

14. Angulo P. Nonalcoholic Fatty Liver Disease. *N Engl J Med.* April 18, 2002 2002;346(16):1221-1231.

15. Michael MD, Kulkarni RN, Postic C, et al. Loss of Insulin Signaling in Hepatocytes Leads to Severe Insulin Resistance and Progressive Hepatic Dysfunction. *Mol Cell.* 2000;6(1):87-97.

16. Biddinger SB, Hernandez-Ono A, Rask-Madsen C, et al. Hepatic Insulin Resistance Is Sufficient to Produce Dyslipidemia and Susceptibility to Atherosclerosis. *Cell Metabolism.* 2008;7(2):125-134.

17. Brown MS, Goldstein JL. Selective versus Total Insulin Resistance: A Pathogenic Paradox. *Cell Metabolism.* 2008;7(2):95-96.

18. Griffin ME, Marcucci MJ, Cline GW, et al. Free fatty acid-induced insulin resistance is associated with activation of protein kinase C theta and alterations in the insulin signaling cascade. *Diabetes.* June 1, 1999 1999;48(6):1270-1274.

19. Lanning N, Carter-Su C. Recent advances in growth hormone signaling. *Reviews in Endocrine & Metabolic Disorders.* 2006;7(4):225-235.

20. Dominici FP, Argentino DP, Muñoz MC, Miquet JG, Sotelo AI, Turyn D. Influence of the crosstalk between growth hormone and insulin signalling on the modulation of insulin sensitivity. *Growth Hormone & IGF Research.* 2005;15(5):324-336.

21. Jeffcoate W. Growth Hormone Therapy and its Relationship to Insulin Resistance, Glucose Intolerance and Diabetes Mellitus: A Review of Recent Evidence. *Drug Safety.* 2002;25(3):199-212.

22. Lee H-W, Muniyappa R, Harman SM, Blackman MR. Long-Term Growth Hormone (GH) Administration Increases Hepatic Insulin Resistance, but Not Muscle Insulin Resistance, in Healthy Older Men and Women. *Endocrine Reviews.*32(03_MeetingAbstracts):OR27-24.

23. Thirone ACP, Carvalho CRO, Brenelli SL, Velloso LA, J.A. Saad M. Effect of chronic growth hormone treatment on insulin signal transduction in rat tissues. *Molecular and Cellular Endocrinology.* 1997;130(1-2):33-42.

24. Ghanaat F, Tayek JA. Growth hormone administration increases glucose production by preventing the expected decrease in glycogenolysis seen with fasting in healthy volunteers. *Metabolism.* 2005;54(5):604-609.

25. Schwarz J-M, Mulligan K, Lee J, et al. Effects of Recombinant Human Growth Hormone on Hepatic Lipid and Carbohydrate Metabolism in HIV-Infected Patients with Fat Accumulation. *Journal of Clinical Endocrinology & Metabolism.* February 1, 2002 2002;87(2):942.

26. Johansen T, Laurino C, Barreca A, Malmlöf K. Reduction of adiposity with prolonged growth hormone treatment in old obese rats: effects on glucose handling and early insulin signaling. *Growth Hormone & IGF Research.* 2005;15(1):55-63.

27. White MF. The IRS-signalling system: A network of docking proteins that mediate insulin action. *Molecular and Cellular Biochemistry.* 1998;182(1):3-11.

28. Barbour LA, Mizanoor Rahman S, Gurevich I, et al. Increased P85 Is a Potent Negative Regulator of Skeletal Muscle Insulin Signaling and Induces in Vivo Insulin Resistance Associated with Growth Hormone Excess. *Journal of Biological Chemistry.* November 11, 2005 2005;280(45):37489-37494.

29. del Rincon J-P, Iida K, Gaylinn BD, et al. Growth Hormone Regulation of p85 Expression and Phosphoinositide 3-Kinase Activity in Adipose Tissue: Mechanism for Growth Hormone–Mediated Insulin Resistance. *Diabetes.* June 2007 2007;56(6):1638-1646.

30. Brachmann SM, Ueki K, Engelman JA, Kahn RC, Cantley LC. Phosphoinositide 3-Kinase Catalytic Subunit Deletion and Regulatory Subunit Deletion Have Opposite Effects on Insulin Sensitivity in Mice. *Molecular and Cellular Biology.* March 1, 2005 2005;25(5):1596-1607.

31. Masternak MM, Panici JA, Bonkowski MS, Hughes LF, Bartke A. Insulin Sensitivity as a Key Mediator of Growth Hormone Actions on Longevity. *The Journals of Gerontology Series A: Biological Sciences and Medical Sciences.* May 1, 2009 2009;64A(5):516-521.

32. Mavalli MD, DiGirolamo DJ, Fan Y, et al. Distinct growth hormone receptor signaling modes regulate skeletal muscle development and insulin sensitivity in mice. *The Journal of Clinical Investigation.* 2010;120(11):4007-4020.

33. Vijayakumar A, Wu Y, Sun H, et al. Targeted Loss of GHR Signaling in Mouse Skeletal Muscle Protects Against High-Fat Diet–Induced Metabolic Deterioration. *Diabetes.* January 1, 2012 2012;61(1):94-103.

34. Fan Y, Menon RK, Cohen P, et al. Liver-specific deletion of the growth hormone receptor reveals essential role of growth hormone signaling in hepatic lipid metabolism. *J Biol Chem.* Jul 24 2009;284(30):19937-19944.

35. Sos BC, Harris C, Nordstrom SM, et al. Abrogation of growth hormone secretion rescues fatty liver in mice with hepatocyte-specific deletion of JAK2. *J Clin Invest.* April 1, 2011 2011;121(4).

36. Cui Y, Hosui A, Sun R, et al. Loss of signal transducer and activator of transcription 5 leads to hepatosteatosis and impaired liver regeneration. *Hepatology.* Aug 2007;46(2):504-513.

37. Barclay JL, Nelson CN, Ishikawa M, et al. GH-Dependent STAT5 Signaling Plays an Important Role in Hepatic Lipid Metabolism. *Endocrinology.* January 1, 2011 2011;152(1):181-192.

38. Shi SY, Martin RG, Duncan RE, et al. Hepatocyte-specific Deletion of Janus Kinase 2 (JAK2) Protects against Diet-induced Steatohepatitis and Glucose Intolerance. *Journal of Biological Chemistry.* March 23, 2012 2012;287(13):10277-10288.

39. Wu Y, Liu C, Sun H, et al. Growth hormone receptor regulates cell hyperplasia and glucose-stimulated insulin secretion in obese mice. *The Journal of Clinical Investigation.* 2011;121(6):2422-2426.

40. Lichanska AM, Waters MJ. How growth hormone controls growth, obesity and sexual dimorphism. *Trends Genet.* Jan 2008;24(1):41-47.

41. Møller N, Gjedsted J, Gormsen L, Fuglsang J, Djurhuus C. Effects of growth hormone on lipid metabolism in humans. *Growth Hormone & IGF Research.* 2003;13(Supplement 1):S18-S21.

42. Nam SY, Marcus C. Growth hormone and adipocyte function in obesity. *Hormone Research.* 2000;53 Suppl 1:87-97.

43. Fineberg SE, Merimee TJ. Acute metabolic effects of human growth hormone. *Diabetes.* Jun 1974;23(6):499-504.

44. Brummer RJM. Effects of growth hormone treatment on visceral adipose tissue. *Growth Hormone & IGF Research.* 1998;8, Supplement 2(0):19-23.

45. Randle PJ, Garland PB, Hales CN, Newsholme EA. THE GLUCOSE FATTY-ACID CYCLE ITS ROLE IN INSULIN SENSITIVITY AND THE METABOLIC DISTURBANCES OF DIABETES MELLITUS. *The Lancet.* 1963;281(7285):785-789.

46. Yu C, Chen Y, Cline GW, et al. Mechanism by Which Fatty Acids Inhibit Insulin Activation of Insulin Receptor Substrate-1 (IRS-1)-associated Phosphatidylinositol 3-Kinase Activity in Muscle. *Journal of Biological Chemistry.* December 27, 2002 2002;277(52):50230-50236.

47. Sherlock M, Woods C, Sheppard MC. Medical therapy in acromegaly. *Nat Rev Endocrinol.* 2011;7(5):291-300.

48. Parkinson C, Trainer PJ. Pegvisomant: a growth hormone receptor antagonist for the treatment of acromegaly. *Growth hormone & IGF research : official journal of the Growth Hormone Research Society and the International IGF Research Society.* Apr 2000;10 Suppl B:S119-123.

49. Parkinson C, Scarlett JA, Trainer PJ. Pegvisomant in the treatment of acromegaly. *Adv Drug Deliv Rev.* Sep 26 2003;55(10):1303-1314.

50. van der Lely AJ, Hutson RK, Trainer PJ, et al. Long-term treatment of acromegaly with pegvisomant, a growth hormone receptor antagonist. *Lancet.* Nov 24 2001;358(9295):1754-1759.

51. Rose DR, Clemmons DR. Growth hormone receptor antagonist improves insulin resistance in acromegaly. *Growth Hormone & IGF Research.* 2002;12(6):418-424.

52. Lindberg-Larsen R, Møller N, Schmitz O, et al. The Impact of Pegvisomant Treatment on Substrate Metabolism and Insulin Sensitivity in Patients with Acromegaly. *Journal of Clinical Endocrinology & Metabolism.* May 1, 2007 2007;92(5):1724-1728.

53. Higham CE, Rowles S, Russell-Jones D, Umpleby AM, Trainer PJ. Pegvisomant improves insulin sensitivity and reduces overnight free fatty acid concentrations in patients with acromegaly. *The Journal of clinical endocrinology and metabolism.* Jul 2009;94(7):2459-2463.

54. Parkinson C, Drake WM, Roberts ME, Meeran K, Besser GM, Trainer PJ. A Comparison of the Effects of Pegvisomant and Octreotide on Glucose, Insulin, Gastrin, Cholecystokinin, and Pancreatic Polypeptide Responses to Oral Glucose and a Standard Mixed Meal. *Journal of Clinical Endocrinology & Metabolism.* April 1, 2002 2002;87(4):1797-1804.

55. Muller AF, Leebeek FWG, Janssen JAMJL, Lamberts SWJ, Hofland L, van der Lely AJ. Acute Effect of Pegvisomant on Cardiovascular Risk Markers in Healthy Men: Implications for the Pathogenesis of Atherosclerosis in GH Deficiency. *Journal of Clinical Endocrinology & Metabolism.* November 1, 2001 2001;86(11):5165-5171.

56. Grinspoon S. Strategies to augment growth-hormone secretion in obesity. *Nat Clin Pract End Met.* 2009;5(3):123-123.

57. Johannsson G, Mårin P, Lönn L, et al. Growth Hormone Treatment of Abdominally Obese Men Reduces Abdominal Fat Mass, Improves Glucose and Lipoprotein Metabolism, and Reduces Diastolic Blood Pressure. *Journal of Clinical Endocrinology & Metabolism.* March 1, 1997 1997;82(3):727-734.

58. Lo JC, Mulligan K, Noor MA, et al. The Effects of Recombinant Human Growth Hormone on Body Composition and Glucose Metabolism in HIV-Infected Patients with Fat Accumulation. *Journal of Clinical Endocrinology & Metabolism.* August 1, 2001 2001;86(8):3480-3487.

59. Kotler DP, Muurahainen N, Grunfeld C, et al. Effects of Growth Hormone on Abnormal Visceral Adipose Tissue Accumulation and Dyslipidemia in HIV-Infected Patients. *JAIDS Journal of Acquired Immune Deficiency Syndromes.* 2004;35(3):239-252.

60. DeFronzo RA, Tobin JD, Andres R. Glucose clamp technique: a method for quantifying insulin secretion and resistance. *American Journal of Physiology - Endocrinology And Metabolism.* September 1, 1979 1979;237(3):E214-223.

61. Rao MN, Mulligan K, Tai V, et al. Effects of Insulin-Like Growth Factor (IGF)-I/IGF-Binding Protein-3 Treatment on Glucose Metabolism and Fat Distribution in Human Immunodeficiency Virus-Infected Patients with Abdominal Obesity and Insulin Resistance. *Journal of Clinical Endocrinology & Metabolism.* September 1, 2010 2010;95(9):4361-4366.

62. Mulligan K, Khatami H, Schwarz J-M, et al. The Effects of Recombinant Human Leptin on Visceral Fat, Dyslipidemia, and Insulin Resistance in Patients with Human Immunodeficiency Virus-Associated Lipoatrophy and Hypoleptinemia. *Journal of Clinical Endocrinology & Metabolism.* April 1, 2009 2009;94(4):1137-1144.

63. Finegood DT, Bergman RN, Vranic M. Estimation of Endogenous Glucose Production During Hyperinsulinemic-Euglycemic Glucose Clamps: Comparison of Unlabeled and Labeled Exogenous Glucose Infusates. *Diabetes.* August 1, 1987 1987;36(8):914-924.

64. Ferrannini E. The theoretical bases of indirect calorimetry: A review. *Metabolism.* 1988;37(3):287-301.

65. Mulligan K, Tai VW, Schambelan M. Effects of Chronic Growth Hormone Treatment on Energy Intake and Resting Energy Metabolism in Patients with Human Immunodeficiency Virus-Associated Wasting—A Clinical Research Center Study. *Journal of Clinical Endocrinology & Metabolism.* May 1, 1998 1998;83(5):1542-1547.

66. Mulligan K, Grunfeld C, Hellerstein MK, Neese RA, Schambelan M. Anabolic effects of recombinant human growth hormone in patients with wasting associated with human immunodeficiency virus infection.

Journal of Clinical Endocrinology & Metabolism. October 1, 1993 1993;77(4):956-962.

67. Wolfe R, Chinkes D. Isotope Tracers in Metabolic Research. Principles and Practice of Kinetic Analysis. 2005. *Hoboken, NJ, Willey and Sons.*

68. Hellerstein MK, Neese RA. Mass isotopomer distribution analysis at eight years: theoretical, analytic, and experimental considerations. *American Journal of Physiology - Endocrinology And Metabolism.* June 1, 1999 1999;276(6):E1146-E1170.

69. Paisley AN, Hayden K, Ellis A, Anderson J, Wieringa G, Trainer PJ. Pegvisomant interference in GH assays results in underestimation of GH levels. *European journal of endocrinology / European Federation of Endocrine Societies.* Mar 2007;156(3):315-319.

70. Jørgensen JOL, Feldt-Rasmussen U, Frystyk J, et al. Cotreatment of Acromegaly with a Somatostatin Analog and a Growth Hormone Receptor Antagonist. *Journal of Clinical Endocrinology & Metabolism.* October 1, 2005 2005;90(10):5627-5631.

71. Matsuda M, DeFronzo RA. I *n vivo* measurement of insulin sensitivity in humans. In: Draznin B, Rizza R, eds. *Methods, assessment, and metabolic regulation.* Vol 1. Totowa, NJ: Humana Press; 1997:23-65.

72. Andreassen M, Frystyk J, Faber J, Kristensen LØ. GH activity and markers of inflammation: a crossover study in healthy volunteers treated with GH and a GH receptor antagonist. *European Journal of Endocrinology.* May 1, 2012 2012;166(5):811-819.

If you have a separate bibliography, attach it to the submission with your other study documents.

11.0 Sample Size and Eligibility

11.1 Number of subjects that will be enrolled at UCSF and affiliated institutions:

6

11.2 Total number of subjects that will be enrolled at all sites (Help Text updated 9/13):

6

11.3 Estimated number of people that you will need to consent and screen here (but not necessarily enroll) to get the needed subjects:

24

11.4 Explain how and why the number of subjects was chosen (Help Text updated 9/13):

This is a pilot study. Enrollment of 12 subjects will provide 80% power by a paired t-test to detect a standardized effect size (mean change from baseline divided by the standard deviation of the change) of 0.9. Because the metabolic assessments are highly sensitive and are performed under carefully controlled conditions, and our focus on changes within each subject automatically controls for inherent differences between subjects, we expect that variability in the within-subject changes will be small enough to allow detection of significant changes using the relatively small sample size appropriate for this proof-of-principle study. We have successfully employed this study design in other proof-of-principle studies testing novel pharmacologic treatments [25,58,61,62,66]. For example, significant changes in insulin sensitivity were seen in one study from our laboratory, in which GH was administered to *six* HIV-positive patients with fat accumulation [58]. Data from that study yielded standardized effect sizes of 1.11 (M/I), 1.15 (EGP), and 2.59 (lipolysis) for the changes during treatment [25,58]. Because all of these values exceed the standardized effect size of 0.9 that we can detect within a group of 12, we expect to have greater than 80% power to detect significant changes in all of these outcomes.

11.5 * Eligible age range(s):

- [ ] 0-6 years
- [ ] 7-12 years
- [ ] 13-17 years
- [x] 18+ years

11.6 Inclusion criteria:

Major inclusion criteria include body mass index >18.5 and <35 kg/m2, insulin resistance as determined by a calculated homeostatic model of insulin resistance (HOMA-IR) score >2.77, normal liver function (ALT and AST < 2X upper limit of normal), ability and willingness to self-administer daily subcutaneous injections, on a stable diet and exercise regimen for at least 4 weeks before screening with plans to remain so throughout the study, and stable weight (+/-5% by self-report) for at least 3 months before screening with agreement to maintain weight during study. Women of childbearing potential may enroll if they are on an approved birth control method. Persons on lipid lowering therapy may enroll if treatment has been stable for at least 3 months.

11.7 Exclusion criteria:

Key exclusion criteria include history of type 1 or type 2 diabetes mellitus, unstable hypertension, pregnancy or breastfeeding within previous 6 months, history of major gastrointestinal surgery, evidence of kidney

disease (serum creatinine >1.7 mg/dL in men and >1.5 mg/dL in women), fasting glucose > 126 mg/dL, HgbA1C > 6.5%, fasting triglycerides >500 mg/dL or history of pancreatitis or liver, biliary, or intestinal disease.

11.8 There are inclusion or exclusion criteria based on gender, race or ethnicity:

○ Yes ◉ No

If **yes**, please explain the nature and rationale for the restrictions:

12.0 Drugs and Devices

12.1 * Investigational drugs or biologics will be used OR approved drugs or biologics will be studied under this application:

◉ Yes ○ No

12.2 * Investigational medical devices or in vitro diagnostics will be used OR approved medical devices or in vitro diagnostics will be studied under this application:

○ Yes ◉ No

12.3 * A Non-Significant Risk (NSR) determination is being requested for an investigational device:

○ Yes ◉ No

12.4 Verification of IND/IDE numbers: If the sponsor's protocol does not list the IND/IDE number, you must submit documentation from the sponsor or FDA identifying the IND/IDE number for this study. Attach this documentation in the Other Study Documents section of the Initial Review Submission Packet.

13.0 Study Drug Details

13.1 List the drugs or biologics that will be studied:

	Drug Name	FDA Approved	A new drug or a new use of approved drug	IND Number
—	Trade Drug Name: SOMAVERT<br>Generic Drug Name: PEGVISOMANT<br>Investigational Drug Name:	Yes	Yes	

Trade Drug Name:	SOMAVERT
Generic Drug Name:	PEGVISOMANT
Investigational Drug Name:	
Identify the name of the manufacturer or source of investigational drug/biologic:	Pfizer
Is the drug supplied at no cost?:	Yes
Is the Drug FDA Approved:	Yes
Is this a new drug or a new use of an already approved drug:	Yes
Is an IND necessary:	No

IND Number:	
Who holds the IND:	N/A
IND details:	
If FDA Approved and an IND is not required, Please provide a rationale for exemption :	
Are you currently using this IND in another research project?:	No
If yes, list the IRB Number(s):Study Number	
Will the investigational pharmacy be dispensing?:	No
If the source is not a FDA licensed facility, provide details regarding the purity, quality, stability and sterility of the investigational drug/biologic:	

14.0 Other Approvals and Registrations

14.1 * Do any study activities take place on patient care units:

○ Yes ◉ No

If **Yes**, attach a letter of support for the study from the involved patient care manager(s).

14.2 * Does your protocol involve any radiation exposure to patients/subjects? The UCSF Radiation Safety Committee requires review of your protocol if it includes administration of radiation as part of standard of care OR research exposures:

◉ Yes ○ No

14.3 * This study may generate genetic data that may be broadly shared (e.g. submitted to NIH for Genome-Wide Association Studies (GWAS) in dbGaP, TCGA, etc):

○ Yes ◉ No

14.4 * This study involves administration of vaccines produced using recombinant DNA technologies to human subjects:

○ Yes ◉ No

14.5 * This study involves human gene transfer (NOTE: Requires NIH Recombinant DNA Advisory Committee (RAC) review prior to CHR approval):

○ Yes ◉ No

14.6 This study involves other regulated materials and requires approval and/or authorization from the following regulatory committees:

☐ Institutional Biological Safety Committee (IBC)

Specify BUA #:

☐ Institutional Animal Care and Use Committee (IACUC)

Specify IACUC #:

☑ Radiation Safety Committee

Specify RUA #:

This study has RSC approval but does not require an RUA

☐ Radioactive Drug Research Committee (RDRC)

Specify RDRC #:

☐ Controlled Substances

15.0 Procedures

15.1 * Procedures/Methods (Help Text updated 9/13) For clinical research list all study procedures, test and treatments required for this study, including when and how often they will be performed. If there are no clinical procedures, describe the Methods:

Screening and Eligibility Assessment: Screening will include a complete medical history and physical examination, and collection of blood samples. Major inclusion criteria include body mass index >18.5 and <35 kg/m2, normal liver function (ALT and AST < 2X upper limit of normal), ability and willingness to self-administer daily subcutaneous injections, on a stable diet and exercise regimen for at least 4 weeks before screening with plans to remain so throughout the study, and stable weight (+/-5% by self-report) for at least 3 months before screening with agreement to maintain weight during study. Women of childbearing potential may enroll if they are on an approved birth control method. Persons on lipid lowering therapy may enroll if treatment has been stable for at least 3 months. Key exclusion criteria include history of type 1 or type 2 diabetes mellitus, unstable hypertension, pregnancy or breastfeeding within previous 6 months, history of major gastrointestinal surgery, evidence of kidney disease (serum creatinine >1.7 mg/dL in men and >1.5 mg/dL in women), fasting glucose > 126 mg/dL, HgbA1C > 6.5%, fasting triglycerides >500 mg/dL or history of pancreatitis or liver, biliary, or intestinal disease.

For screening, the presence of insulin resistance will be determined by the use of the homeostasis model assessment of insulin resistance (HOMA-IR) score derived from the fasting plasma glucose and serum insulin levels. A HOMA-IR level >2.77 will be used for these purposes based on the observation that such a score placed an individual in the uppermost quintile of subjects studied in a large survey of insulin resistance and cardiovascular risk factors. Diabetes will be ruled out by measurement of fasting glucose and HbA1c, and by performance of a 2-hour oral glucose tolerance test. After collection of fasting blood specimens, the subject will drink a solution containing 75 g dextrose. A blood sample will be collected 2 hours later for determination of plasma glucose.

Baseline Inpatient Assessment and Initiation of Treatment: Subjects will be admitted to the CRC in the afternoon of the day their testing begins and spend that night in the CRC. During their admission they will be fed a controlled metabolic diet with fixed proportions of macronutrients (10 kcal/kg; 15% protein, 30% fat, 55% carbohydrate with < 20% from simple sugars). All meals will be prepared in the metabolic kitchen of the CRC under the supervision of CRC bionutritionists. Women of childbearing potential will undergo a urine pregnancy test at the time of admission.

Whole-body EGP and lipolysis will be assessed by simultaneous infusion of labeled glucose and glycerol both under fasting conditions and during hyperinsulinemia. We will also perform dual-energy X-ray absorptiometry (DXA) scanning to assess total and regional lean and fat distribution.

On the evening of their admission, subjects will receive a fixed metabolic meal. In reparation for the infusions, intravenous lines will be placed and baseline samples drawn. All infusions will be given via a single venous catheter. At 4:30 AM the following morning, primed infusions of [1- ^{13}C] glucose (0.4 mmol/kg/min) and [d $_5$] glycerol (9.7 mg/kg/min) will begin. Blood samples will be collected at 8:00, 8:10, 8:20, and 8:30 AM for fasting steady-state measurements. The isotope infusions will continue, and at 9:00 AM a euglycemic-hyperinsulinemic clamp will be started [58,60-62]. After collection of fasting blood samples for measurement of lipids (TG, total, HDL, and LDL cholesterol, FFA), IGF-I, insulin, and GH, a primed infusion of insulin (20 mU/m ^{2}/min; bound to human albumin) will administered for 3 hours. In previous studies using this dose of insulin we have achieved steady-state serum insulin levels in the range of 80-100 µIU/mL, which have been sufficient to show effects on both peripheral glucose uptake and EGP [58,61,62].Blood samples will be collected at 5-minute intervals from a retrograde intravenous line placed in a hand that is warmed in a heated box at 50-55 ^{o} C. Whole-blood glucose concentrations will be determined at 5-minute intervals using a YSI STAT 2300 glucose analyzer, and an infusion of 20% dextrose (labeled with 6% [6,6d2] glucose will be adjusted to maintain euglycemia [63]. Additional blood samples will be collected at half-hour intervals for

measurement of serum insulin concentrations and at 11:30, 11:40, 11:50 AM, and 12:00 PM for steady state isotopic measurements during hyperinsulinemia. At 12:00 PM, the clamp will be discontinued, as will the isotopic glucose and glycerol infusions. Subjects will be fed a full lunch upon completion of the clamp and discharged after their first injection of study medication (see below).

Resting Energy Expenditure (REE) and Substrate Oxidation Rates: To evaluate the effects of GHR antagonism on REE and substrate oxidation rates, we will perform indirect calorimetry before and during the clamp using a ventilated canopy system(Parvomedics Metabolic Monitor, Sandy UT). Net lipid oxidation will be calculated using stoichiometrically-derived equations [64]. In previous studies we have demonstrated that treatment with pharmacologic doses of GH increased REE and lipid oxidation [25,65,66], consistent with its lipolytic effects.

Pegvisomant Treatment: Upon completion of baseline testing, subjects will begin open-label treatment with recombinant human pegmisovant in a dose of 20 mg, which will be administered by daily subcutaneous injection. Treatment will occur at bedtime. CRC nurses will provide instruction on proper technique for self-administering subcutaneous injections and observe the subjects giving themselves their first injection. A one-week supply of study medication and supplies will be dispensed.

Follow-up Studies: Subjects will return to the CRC at weekly intervals after initiation of study medication for safety assessments. During these visits, their medical history will be updated and a brief physical exam, including weight and vital signs, will be performed. All reported signs and symptoms will be recorded. Safety laboratory studies (i.e. fasting glucose, lipids, electrolytes, and renal and hepatic function) as well as insulin, GH and IGF1 levels will be obtained reviewed on a continuous basis by the investigator. At these visits they will also return unused vials of drug and will receive their next week's supply of drug and supplies.

Final Inpatient Assessment: After 4 weeks of outpatient treatment, subjects will be readmitted to the CRC to repeat all of the assessments performed at baseline. During this admission, they will be fed the same diet as during the first admission and they will receive their final dose of study medication during the admission. All unused medication and supplies will be returned to the investigators. Treatment will be discontinued.

Laboratory Methods:

Stable Isotope Determinations: Stable isotope analyses will be performed using gas chromatography/mass spectrometry in the Endocrine Division Stable Isotope Laboratory at SFGH, as used in previous studies by our group [25,61,62]. The rates of appearance (Ra) of glucose and glycerol (lipolysis) will be calculated using standard dilution principles [67].

Miscellaneous Assays: Bedside measurement of whole-body glucose during the euglycemic hyperinsulinemic clamp will be determined by the glucose oxidase method, using a YSI STAT 2300 Glucose Analyzer (Yellow Springs, OH). Plasma TG , FFA, and total, HDL, and direct LDL cholesterol levels will be measured in a commercial laboratory. Serum insulin will be measured in the PI's laboratoryusing reagents from Linco/Millipore (St. Charles, MO). Serum IGF1will be measured using a standard commercially available assay. The measurement of GH in patients on pegvisomant is complex [69]. Therefore, GH will be measured using the previously published DELFIA method which permits specific detection of GH and distinguishes from pegvisomant [70]. Glycosylated hemoglobin and fructosamine will be measured in the SFGH Clinical Laboratories.

Peripheral glucose uptake during the clamp (M-value) will be calculated to reflect the contributions of both exogenous glucose (infusion rate of 20% dextrose) and endogenous glucose (EGP) during the final hour of the clamp and will be adjusted for steady-state insulin levels (M/I) [60,71].

If you have a procedure table, attach it to the submission with your other study documents.

15.2 Interviews, questionnaires, and/or surveys will be administered or focus groups will be conducted:

○ Yes ◉ No

List any standard instruments used for this study:

Attach any non-standard instruments at the end of the application.

15.3 Conduct of study procedures or tests off-site by non-UCSF personnel:

○ Yes ◉ No

If yes, explain:

15.4 Sharing of experimental research test results with subjects or their care providers:

○ Yes ◉ No

If yes, explain:

15.5 * Specimen collection for future research and/or specimen repository/bank administration:

◉ Yes ○ No

15.6 Time commitment (per visit and in total):

The screening visit should require approximately 3 hours. Both inpatient admissions will last approximately 24 hours. The follow-up visits at the end of weeks 1, 2 and 3 should require approximately 2 hours. Total time: 57 hours.

15.7 Locations:

All inpatient and outpatient visits will take place in the CTSI Clinical Research Center at SFGH, which is a physically discrete multicategorical clinical research facility located in the main hospital building. The facility consists of 10 inpatient beds, 5 outpatient exam rooms, all of the equipment that is required for this study (e.g. infusion pumps), a fully equipped metabolic kitchen, and facilities for processing and short-term storage of biologic specimens. There is 24-hour nursing coverage in the unit. The nursing staff of the SFGH-CRC has had considerable experience in supporting the types of metabolic studies outlined in this application.

15.8 Describe the resources in place to conduct this study in a way that assures protection of the rights and welfare of participants:

All studies will be performed in the CRC at SFGH. Nursing staff is available at all times. Should a medical emergency arise, the PI and Co-I would be contacted immediately and appropriate treatment initiated, including transfer to the emergency department if necessary.

16.0 Specimen Collection for Future Research and/or Specimen Repository/Bank Administration

16.1 Specimens are (check all that apply):

☐ Surplus clinical specimens from a diagnostic or therapeutic procedure
☑ Specimens collected for research purposes only

☐ Other

If Other, explain:

16.2 Types of specimens:

☑ Blood
☐ Tissue (describe below):
☐ Existing/archival materials (name source below): --
☐ Other (describe below):

Describe and/or name source:

16.3 Consent will be obtained via:

☐ Separate specimen banking consent form
☑ Specimen banking section within a main research study consent form
☐ Surgical consent form with tissue donation brochure

16.4 Specimens will ultimately be stored (check all that apply):

UCSF

☑ UCSF repository/bank being established under this protocol
☐ Existing UCSF specimen repository/bank with CHR approval

Provide the name of the bank and CHR approval number (if not being banked at UCSF under this protocol):

Outside Entity

☐ Cooperative group bank
☐ NIH
☐ Other university
☐ Industry sponsor
☐ Other

Specify to what institution, cooperative group or company specimens will be transferred:

16.5 Direct identifiers will be sent with specimens or shared with other researchers and/or outside entities:

○ Yes
◉ No
○ N/A - Specimens will not be shared with others

If
Yes
, which identifiers will be sent with specimens:

☐ Name
☐ Date of birth
☐ Social Security number
☐ Medical record number
☐ Address
☐ Phone number
☐ Email address
☐ Other dates (surgery date, clinic visit dates, etc.)

If
Yes
, provide a justification for sending direct identifiers with the specimens:

17.0 Establishing a Specimen Repository/Bank at UCSF

17.1 The repository/bank is physically located at (list the address and room number for all locations):

SCVRB 382

17.2 Methods for maintaining confidentiality:

- [x] Samples are completely de-identified before being added to the bank/repository. There is no way to link the specimens back to the subjects.
- [ ] Samples are coded and researchers are able to link the specimens to specific subjects. However, future recipients will not receive direct identifiers with the specimens.
- [ ] Samples are stored with direct identifiers in the repository. However, future recipients will not receive direct identifiers with the specimens.
- [ ] Samples are coded and/or kept with direct identifiers in the repository. The bank/repository may release identifiers with specimens to researchers under special circumstances with prior IRB approval.

Explain under what circumstances identifiers may be released:

17.3 If the repository/bank maintains the identifiers, list the identifiers that will be maintained with the specimens:

- [ ] Name
- [ ] Date of birth
- [ ] Social Security number
- [ ] Medical record number
- [ ] Address
- [ ] Phone number
- [ ] Email address
- [ ] Other dates (dates of surgery, visit dates)

17.4 Clinical follow-up data will be linked to specimens:

○ Yes ● No

If
Yes
, provide duration of follow-up or indefinitely:

17.5 There is a formal specimen utilization review process:

○ Yes ● No

If
Y

es
, describe the process:

17.6 Specimens banked at UCSF may be made available to (check all that apply):

- [x] UCSF researchers
- [ ] Non-UCSF researchers

☐ Industry

18.0 Alternatives

18.1 Study drug or treatment is available off-study:

○ Yes
◉ No
○ Not applicable

18.2 * Is there a standard of care (SOC) or usual care that would be offered to prospective subjects at UCSF (or the study site) if they did not participate:

○ Yes ◉ No

If yes, describe the SOC or usual care that patients would receive if they choose not to participate:

18.3 Describe other alternatives to study participation that are available to prospective subjects:

Alternatives include standard treatment for insulin resistance, including diet, exercise and in some cases, treatment with metformin.

19.0 Risks and Benefits

19.1 * Risks and discomforts:

Pegvisomant treatment:Since pegvisomant is an approved drug with a long track record of safety data, we feel that the study as designed is safe. We do not anticipate any significant toxicity of the drug. There have been limited descriptions of local lipodystrophy at the site of injection, but these have all been short-lived. We do not anticipate any adverse effects in terms of hypoglycemia. In patients with diabetes and acromegaly, hypoglycemia is an ongoing concern and patients often have to adjust their diabetes drugs. However, as described above, we have chosen to exclude patients currently on diabetes drugs. In addition, we will closely monitor fasting glucose levels and other potential effects both during and for 4 weeks following treatment.

Glucose/Insulin Clamp: Infusion of insulin and glucose may cause a burning sensation in the arm that is receiving the infusion or symptoms of hypoglycemia or hyperglycemia. The infusion study will be discontinued of any of these symptoms occur. Frequent monitoring of blood glucose will allow the recognition and prevention of significant hypo- or hyperglycemia.

Worsening Glucose Homeostasis: Although we expect, if anything, to see improvements in glucose homeostasis with pegvisomant, we cannot exclude the possibility that glucose homeostasis may deteriorate during the course of the study. Should such occur, we will consult with the primary physician, who will decide whether pharmacologic treatment is indicated. If a subject discontinues study medication during the treatment period, we will continue to follow him/her for safety purposes, and their data will be included as part of the safety analysis.

Risks of phlebotomy:The withdrawal of blood for the entire study will total no more than 440mL. This amount is within allowable limits for adults and should not lead to anemia or hypotension in the population we will study.

Venipuncture and placement of intravenous linesmay result in hematoma, which can be painful but carries no significant risks. Infections from IV lines rarely occur from such brief placements. However, any signs of inflammation will lead to removal of the catheter and treatment with heat and elevation.

Radiation exposure: Exposure from DXA is less than the yearly natural background radiation in the US, which is 3 mSv and is considered to involve minimal risk. Participants will be informed about radiation exposure and will be queried about other recent X-ray exposure so that cumulative X-ray exposure will not be excessive. Pregnancy testing in women of childbearing potential will be performed before DXA scanning.

Stable isotopes:The stable isotopes of 2H contain no radioactivity and have no recognized adverse effects at the doses given. Stable isotope stock solutions will be prepared by the investigational pharmacy at UCSF, where they will be tested for sterility and pyrogenicity and aliquotted into single-use vials. Intravenous infusion bags will be prepared under sterile conditions.

Hospitalization for 1 day:Participants may experience boredom from confinement on a metabolic ward. The CRC is equipped with computers and other forms of entertainment, as well as wireless internet access, as means of mitigating this risk.

Loss of confidentiality is an unlikely but possible risk. Researchers keep personal and medical data confidential. All information is considered confidential including answers to questions, physical exams, and findings on both images and blood tests. The relative health of the participants and the nature of this study make it unlikely that a loss of confidentiality would lead to any psychological or social harm.

19.2 Steps taken to minimize risks to subjects:

The specific measures to minimize each risk are described in the Discussion of Risks, as well as the Data Safety and Monitoring Plan. In addition, there will be continuous safety surveillance with emphasis on the potential side effects of the drug and or each procedure. Participation in the study will be discontinued if the participant fails to adhere to the study requirements in a way that may cause harm to him or herself or seriously interfere with the validity of the study results; or the investigator determines that further participation would be detrimental to the participant’s health or wellbeing.

Further protection is provided by the fact that the CRC nurses are all trained in metabolic investigations, including placement and maintenance of intravenous lines. All specimens are labeled with a study identification number that can only be linked to the study participant’s identifying information through a key that is maintained by the PI. In the unlikely event that an injury or adverse event should occur during the study, these events will be managed and reported in accordance with the policies of the IRB of UCSF, as explained in the consent form.

19.3 Benefits to subjects:

◉ Yes ○ No

If yes, describe:

The participants may benefit from the knowledge we gain in understanding how GHR antagonism affects insulin/glucose metabolism. However, this benefit cannot be guaranteed. The risks discussed are reasonable in relation to the benefit.

19.4 Benefits to society:

Benefits to society may include a better understanding of the role of GHR antagonism on insulin sensitivity in humans. This could eventually lead to a novel treatment incorporating GHR blockade (or even downstream blockade) as a means to treat or prevent type 2 diabetes. There is considerable controversy surrounding the potential role of GH in carbohydrate metabolism and indeed some investigators are examining the potential benefits of GH treatment on body composition and cardiovascular health (despite the known deleterious effects on insulin/glucose metabolism). This information may provide the basis for understanding the true role of GH signaling and may resolve some incredibly important outstanding questions.

19.5 Explain why the risks to subjects are reasonable:

Pegvisomant is an approved drug with a good safety track record. We feel the risks are reasonable and minimal.

20.0 Data and Safety Monitoring Plan

20.1 Describe the plan for monitoring data and safety (Help Text updated 9/13):

Assessment of Risk:This study presents a moderate degree of risk to the study participants. The study drug is an approved drug available by prescription and has been on the market for greater than 10 years. The drug is generally safe with the most common toxicity being a short-lived local lipodystrophy. The stable isotope studies are complex. No procedure by itself is particularly risky but the performance of multiple procedures and confinement in the CRC for one day lead to an assignment of moderate risk.

Anticipated Adverse Events:The possible side effects and risks of the study procedures are described previously in the above section titled “Risk to the Subjects.”

Safety Monitoring:The studies will be conducted under the medical supervision of Drs. Ethan Weiss and Morris Schambelan, the CRC nurses, and other study personnel. Potential side effects of the treatment and study procedures will be assessed. Safety laboratory studies will be obtained at screening and upon both admissions to the CRC.

Frequency of Safety Reviews :The condition of each participant will be monitored continuously during the inpatient and weekly during the outpatient studies.

Person to Perform Safety Reviews:Dr. Schambelan will have responsibility for the clinical management of participants during their inpatient stays. Safety reviews for all participants will be performed under Dr. Schambelan’s supervision.

Adverse Event Grading Scale:Adverse events will be graded using the following scale:

Grade 1 **Mild** Transient of mild discomfort; no limitation in activity; no medical intervention/therapy required.
Grade 2 **Moderate** Mild to moderate limitation in activity – some assistance may be needed; no medical intervention/therapy required.
Grade 3 **Severe** Marked limitation in activity, some assistance usually required; medical intervention/therapy required, hospitalizations possible.
Grade 4 **Life-threatening** Extreme limitation in activity, significant assistance required; significant medical intervention/s required, hospitalization or hospice care probable.

Reporting of Adverse Events:Reporting of adverse events will follow requirements put forth by the IRB at UCSF. The medical director of the CRC will also be notified should a serious adverse event occur during the inpatient studies.

Study Termination Criteria:If subjects experience a serious adverse effect of pegvisomant, they will be terminated from the study. If the participant is unable to follow through on the blood collection, that inability would constitute adequate reason for study termination. In addition, consistent non-adherence to the drug treatment or refusal to cooperate with study personnel or CRC staff would warrant discontinuation.

20.2 This study requires a Data and Safety Monitoring Board:

○ Yes
◉ No or not sure

If
yes
, press

SAVE and CONTINUE
to move to the next section of the application.

20.3 If No, provide rationale:

- ( ) Social/Behavioral research
- (•) Phase I trial
- ( ) Treatment IND/Compassionate Use Trial
- ( ) Other (explain below)

If
Other,
explain:

This is an open-label study of an FDA-approved drug. Safety monitoring will occur continuously during treatment.

21.0 Confidentiality and Privacy

21.1 Plans for maintaining privacy in the research setting:

Prior to the inpatient admission to the CRC, all subject information will be kept in a research chart (not part of the medical record) that will be stored in a secure place in the PI's office. Laboratory data may only be accessed from the SFGH electronic medical record by care providers with current passwords and appropriate "need to know." During the CRC admissions, all clinical data pertaining to the subject will be kept secure in the CRC subject chart and research data in files kept by the investigator.

21.2 Possible consequences to subjects resulting from a loss of privacy:

Since all subjects in the study will have insulin resistance, participation in the study may identify them as such. Screening and participation in the study are voluntary. During medical interviews, subjects are asked to provide information on alcohol and drug use that might affect the outcome of the study or the subject's ability to adhere to the protocol. Screening and safety laboratory evaluations and can be accessed from the SFGH electronic medical record.

21.3 Study data are:

- [ ] Derived from the Integrated Data Repository (IDR) or The Health Record Data Service (THREDS) at SFGH
- [x] Derived from a medical record (e.g. APeX, OnCore, etc. Identify source below)
- [x] Added to the hospital or clinical medical record
- [x] Created or collected as part of health care
- [x] Used to make health care decisions
- [x] Obtained from the subject, including interviews, questionnaires
- [ ] Obtained from a foreign country or countries only
- [ ] Obtained from records open to the public
- [x] Obtained from existing research records
- [ ] None of the above

If
derived from a medical record
, identify source:

SFGH Medical Record

21.4 Identifiers may be included in research records:

(•) Yes ( ) No

If **yes**, check all the identifiers that may be included:

- [x] Names
- [x] Dates
- [x] Postal addresses
- [x] Phone numbers
- [x] Fax numbers
- [x] Email addresses
- [x] Social Security Numbers*
- [x] Medical record numbers
- [ ] Health plan numbers
- [ ] Account numbers
- [ ] License or certificate numbers
- [ ] Vehicle ID numbers
- [ ] Device identifiers or serial numbers
- [x] Web URLs
- [ ] IP address numbers
- [ ] Biometric identifiers
- [ ] Facial photos or other identifiable images
- [x] Any other unique identifier

* Required for studies conducted at the VAMC

21.5 Identifiable information might be disclosed as part of study activities:

(•) Yes ( ) No

If **yes**, indicate to whom identifiable information may be disclosed:

- [x] The subject's medical record
- [x] The study sponsor
- [ ] Collaborators
- [x] The US Food & Drug Administration (FDA)
- [x] Others (specify below)
- [ ] A Foreign Country or Countries (specify below)

If
Others
, specify:

The subject's primary care doctor with advance permission from the subject.

21.6 Indicate how data are kept secure and protected from improper use and disclosure (check all that apply):
NOTE: Whenever possible, do not store subject identifiers on laptops, PDAs, or other portable devices. If you collect subject identifiers on portable devices, you MUST encrypt the devices.

- [x] Data are stored securely in My Research
- [ ] Data are coded; data key is destroyed at end of study
- [ ] Data are coded; data key is kept separately and securely
- [x] Data are kept in a locked file cabinet
- [x] Data are kept in a locked office or suite
- [x] Electronic data are protected with a password
- [x] Data are stored on a secure network
- [ ] Data are collected/stored using REDCap or REDCap Survey

☐ Data are securely stored in OnCore

21.7 Additional measures to assure confidentiality and protect identifiers from improper use and disclosure, if any:

No names or other identifiers will be used in any publications or reports from this study.

21.8 This study may collect information that State or Federal law requires to be reported to other officials or ethically requires action:

○ Yes ◉ No

Explain:

21.9 This study will be issued a Certificate of Confidentiality:

○ Yes ◉ No

22.0 Subjects

22.1 Check all types of subjects that may be enrolled:

☑ Inpatients
☑ Outpatients
☐ Healthy volunteers
☐ Staff of UCSF or affiliated institutions

22.2 Additional vulnerable populations:

☐ Children
☐ Subjects unable to consent for themselves
☐ Subjects unable to consent for themselves (emergency setting)
☐ Subjects with diminished capacity to consent
☑ Subjects unable to read, speak or understand English
☐ Pregnant women
☐ Fetuses
☐ Neonates
☐ Prisoners
☐ Economically or educationally disadvantaged persons
☐ Investigators' staff
☐ Students

Explain why it is appropriate to include the types of subjects checked above in this particular study:

Although we do not specifically select for them, some participants may be economically or educationally disadvantaged. Economic status is not an exclusion criterion for this protocol, and thus they may enroll in the study if they are willing and qualified. There is no reason to deny such individuals the opportunity to participate in this study.

Describe the additional safeguards that have been included in the study to protect the rights and welfare of these subjects and minimize coercion or undue influence:

Subject payment is such to minimize coercion for the financially disadvantaged while remaining fair based on the requirements of the study protocol, based on similar research previously performed. All subjects are given an in-person review of what is involved and required for the study by staff during the consent process. Questions are

encouraged and feedback is sought to insure that the subject understands what is required and is not influenced for other reasons. Finally, for all participants, the investigators will continue to check with subjects throughout the study to assure their continued understanding of study procedures and requirements and consent to participate.

23.0 Inclusion of Non-English Speaking Subjects

23.1 Indicate which method(s) you will use to consent non-English speaking subjects:

☑ Preferred Method—Consent form and other study documents will be available in the subject's primary language Personnel able to discuss participation in the patient's language will be present for the consent process.

☐ Short-Form—A qualified interpreter will translate the consent form verbally, and subjects will be given the Experimental Subject's Bill of Rights in their primary language, following instructions in Those Who do not Read, Speak or Understand English for required witnessing and signatures

23.2 Explain how you will maintain the ability to communicate with non-English speakers throughout their participation in the study:

Once we receive CHR approval for the English version of the consent, it will be translated to Spanish and Chinese. We anticipate that the vast majority of potential non English-speaking subjects would speak one of these languages. Members of the study staff, CRC nursing staff who are fluent in Spanish or Cantonese, or SFGH interpreters will assist with consent and interacting with study volunteers throughout the study. For participants who speak Mandarin or other Chinese dialects we will use interpreters that are provided by SFGH.

24.0 Recruitment

24.1 * Methods (check all that apply):

☑ Study investigators (and/or affiliated nurses or staff) recruit their own patients directly in person or by phone.

☐ Study investigators recruit their own patients by letter. Attach the letter for review.

☐ Study investigators send a "Dear Doctor" letter to colleagues asking for referrals of eligible patients. If interested, the patient will contact the PI or the PI may directly recruit the patients (with documented permission from the patient). Investigators may give the referring physicians a study information sheet for the patients.

☐ Study investigators provide their colleagues with a "Dear Patient" letter describing the study. This letter can be signed by the treating physicians and would inform the patients how to contact the study investigators. The study investigators may not have access to patient names and addresses for mailing

☑ Advertisements, notices, and/or media used to recruit subjects. Interested subjects initiate contact with study investigators. Attach ads, notices, or media text for review. In section below, please explain where ads will be posted.

☐ Study investigators identify prospective subjects through chart review. (Study investigators request a Waiver of Authorization for recruitment purposes.)

☐ Large-scale epidemiological studies and/or population-based studies: Prospective subjects are identified through a registry or medical records and contacted by someone other than their personal physician. (Study investigators request a Waiver of Authorization for recruitment purposes.)

☑ Direct contact of potential subjects who have previously given consent to be contacted for participation in research. Clinic or program develops a CHR-approved recruitment protocol that asks patients if they agree to be contacted for research (a recruitment database) or consent for future contact was documented using the consent form for another CHR-approved study.

☑ Study investigators list the study on the School of Medicine list of UCSF Clinical Trials website or a similarly managed site. Interested subjects initiate contact with investigators.

☑ Study investigators recruit potential subjects who are unknown to them through methods such as snowball sampling, direct approach, use of social networks, and random digit dialing.

☐ Other

If
Other
, explain:

24.2 * How, when, and by whom eligibility will be determined:

Eligibility will be determined by review of medical charts, if available, pre-screening interviews and in-person assessments carried out by qualified study staff and verified by the Principal Investigator and Co-Investigators.

24.3 * How, when, where and by whom potential subjects will be approached:

We expect that prospective subjects will be referred to us from the Cardiology, Lipid, and general medicine clinics at both SFGH and UCSF. We will also distribute an information sheet to other potential referring physicians in the community who see patients with insulin resistance. A sample recruitment letter with an information sheet is included with this application. We will also post advertisements in clinics at SFGH and UCSF, with Craig's list, and other Internet, social media, and print media. A copy of a sample advertisement is included with this application.

24.4 * Protected health information (PHI) will be accessed prior to obtaining consent:

(•) Yes ( ) No

25.0 Waiver of Consent/Authorization for Recruitment Purposes This section is required when study investigators (and/or affiliated nurses or staff) recruit their own patients directly.

25.1 * Study personnel need to access protected health information (PHI) during the recruitment process and it is not practicable to obtain informed consent until potential subjects have been identified:

(•) Yes

If
no
, a waiver of consent/authorization is NOT needed.

25.2 * A waiver for screening of health records to identify potential subjects poses no more than minimal risk to privacy for participants:

(•) Yes

If
no
, a waiver of authorization can NOT be granted.

25.3 * Screening health records prior to obtaining consent will not adversely affect subjects' rights and welfare:

(•) Yes

If
no
, a waiver of authorization can NOT be granted.

25.4 * Check all the identifiers that will be collected prior to obtaining informed consent:

[x] Names

- [x] Dates
- [x] Postal addresses
- [x] Phone numbers
- [x] Fax numbers
- [x] Email addresses
- [x] Social Security Numbers*
- [x] Medical record numbers
- [ ] Health plan numbers
- [ ] Account numbers
- [ ] License or certificate numbers
- [ ] Vehicle ID numbers
- [ ] Device identifiers or serial numbers
- [x] Web URLs
- [ ] IP address numbers
- [ ] Biometric identifiers
- [ ] Facial photos or other identifiable images
- [x] Any other unique identifier
- [ ] None

Note: HIPAA rules require that you collect the minimum necessary.

25.5 * Describe any health information that will be collected prior to obtaining informed consent:

In a pre-screening telephone interview, we collect only information that will allow us to determine whether we should invite the participant to come to SFGH to undergo in-person screening. This information addresses basic issues of eligibility, such as age, current and previous treatments for diabetes, willingness to participate in a study that requires inpatient admissions and daily medication.

Note: HIPAA requires that you collect the minimum necessary.

25.6 * Describe your plan to destroy the identifiers at the earliest opportunity consistent with the research or provide a health or research justification for retaining the identifiers, or indicate and explain that retention is required by law:

Sensitive documents will be shredded once all data have been collected and reviewed by the investigators.

26.0 Informed Consent

26.1 * Methods (check all that apply):

- [x] Signed consent will be obtained from subjects and/or parents (if subjects are minors)
- [ ] Verbal consent will be obtained from subjects using an information sheet or script
- [ ] Electronic consent will be obtained from subjects via the web or email
- [ ] Implied consent will be obtained via mail, the web or email
- [ ] Signed consent will be obtained from surrogates
- [ ] Emergency waiver of consent is being requested for subjects unable to provide consent
- [ ] Informed consent will not be obtained

26.2 * Process for obtaining informed consent:

At the beginning of the screening visit, a member of the research staff will review the basic and supplementary HIPAA consent forms in detail with the subject and answer all

questions before inviting the subject to sign the consent forms. A photocopy of the signed consent forms with the Experimental Subject's Bill of Rights will be given to the subject.

26.3 * How investigators will make sure subjects understand the information provided to them:

We will ask questions during the consent process and throughout the study to make sure the subject has a good understanding of the study.

27.0 Financial Considerations

27.1 Subjects payment or compensation method (check all that apply):

Payments will be (check all that apply):

- [ ] Subjects will not be paid
- [ ] Cash
- [x] Check
- [ ] Debit card
- [ ] Gift card
- [ ] Reimbursement for parking and other expenses
- [ ] Other:

Specify **Other** :

27.2 Describe the schedule and amounts of payments, including the total subjects can receive for completing the study. If deviating from recommendations in Subject Payment Guidelines, include specific justification below.

Subjects will be paid a total of $1,000 for completing the study. This amount is comparable to payment for other studies that require inpatient metabolic assessments. Should a subject withdraw before completing the study, payment would be prorated as follows: screening $25; first inpatient admission $300; week 1 visit $125; week 2 visit $125; week 3 visit $125; final inpatient visit $300. Payment will be by check once the subject completes the study. Subjects will be advised that they are responsible for reporting this payment as income to the IRS.

27.3 Costs to Subjects: Will subjects or their insurance be charged for any study procedures?

○ Yes ◉ No

If **yes,** describe those costs below, and compare subjects' costs to the costs associated with alternative care off-study. Finally, explain why it is appropriate to charge those costs to the subjects.

28.0 CTSI Screening Questions

28.1 * This study will be carried out at one of the UCSF Clinical Research Services (CRS) centers or will utilize CRS services. CRS centers are at the following sites: SFGH Clinical Research Center Moffitt Adult Clinical Research Center Moffitt Hospital Pediatrics & NCRC Mount Zion Hospital Clinical Research Center Tenderloin Center CHORI Children's Hospital Pediatrics & Adult Clinical Research Center Kaiser Oakland Research Unit SF VA Medical Center Clinical Research Unit Please note: Effective 3/1/14, the CRS form will no longer be completed and

submitted in iRIS. The CRS budget request form can be found at: https://accelerate.ucsf.edu/files/crs/BudgetRequest2015.docx. Follow the instructions on the form to submit. Even if you click 'Yes' to this question, the form will no longer proceed to the Clinical Research Services (CRS) Application Form section.

(•) Yes ( ) No

28.2 This project involves community-based research:

( ) Yes (•) No

28.3 This project involves practice-based research:

( ) Yes (•) No

29.0 End of Study Application

29.1 End of Study Application Form To continue working on the Study Application: Click on the section you need to edit in the left-hand menu. Remember to save through the entire Study Application after making changes. If you are done working on the Study Application: Click Save and Continue. If this is a new study, you will automatically enter the Initial Review Submission Packet form, where you can attach consent forms or other study documents. Review the Initial Review Submission Checklist for a list of required attachments. Answer all questions and attach all required documents to speed up your approval.
