# Supplementary material for: Growth hormone receptor antagonism with pegvisomant in insulin resistant non-diabetic men: A phase II pilot study
Source: F1000Res. 2017 May 3;6:614. [Version 1] doi: 10.12688/f1000research.11359.1 (PMC5499778; doi:10.12688/f1000research.11359.1)
Supplement: Supplementary file 4 [file f1000research-6-12263-s0003.tgz › ace25ef3-71a8-47ec-832f-0efa718690c0.docx]

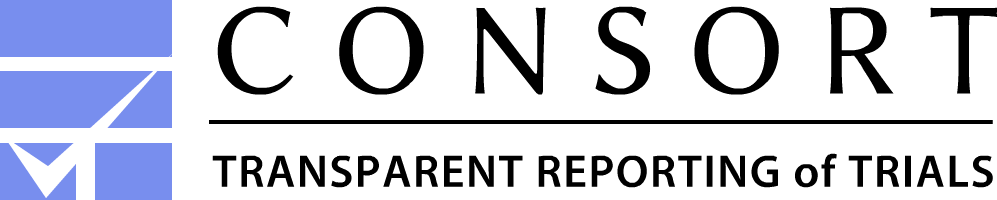


**CONSORT 2010 Flow Diagrams**

Analysed (n= 4)
♦ Excluded from analysis (give reasons) (n= 2). Two participants were excluded from the final analysis because of deviations from the original protocol.

Excluded (n= 205)

♦  Not meeting inclusion criteria (n= 179)

♦  Declined to participate (n=21)

♦  Other reasons (n=5)

## Enrollment

## Analysis

Lost to follow-up (give reasons) (n= 0)

Discontinued intervention (give reasons) (n= 0)

## Allocation

Allocated to intervention (n= 6)

♦ Received allocated intervention (n= 6)

♦ Did not receive allocated intervention (give reasons) (n= 0)

## Follow-Up

Assessed for eligibility (n=211)
