# Supplementary material for: Growth hormone receptor antagonism with pegvisomant in insulin resistant non-diabetic men: A phase II pilot study
Source: F1000Res. 2017 May 3;6:614. [Version 1] doi: 10.12688/f1000research.11359.1 (PMC5499778; doi:10.12688/f1000research.11359.1)
Supplement: Peer review history

# Peer review: Referee response for version 1

**DOI:** 10.5256/f1000research.12263.r23283

**Published:** 2017-07-03

**Authors:** Stuart J Frank, T. Brooks Vaughan

**Affiliations:** 1 Division of Endocrinology, Diabetes and Metabolism, University of Alabama at Birmingham, Birmingham, AL, USA

1. Yes, the current literature is cited. The discussion points out that acromegaly is associated with insulin resistance and Laron dwarfism is associated with insulin sensitivity. While both are true, growth hormone deficiency is also associated with insulin resistance, which may be a state that is more analogous to partial GH receptor blockade which would be induced by pegvisomant (Johannson *et al*, Metabolism 44:1126, 1995 and Alford FP, J Endocrinol Invest 22: 28, 1999, among other sources).
2. The study design is appropriate and conventional means of assessing insulin sensitivity are used.
3. Generally sufficient details of methods and analysis are used. It would be useful to know the age adjusted normal ranges of IGF-1 levels for the subjects in the study to better understand their levels compared to a normal population. Subjects were asked to continue there normal levels of activity, it would be useful to know more about this since exercise can dramatically affect insulin sensitivity.
4. The statistical analysis and interpretation appears to be appropriate.
5. The appropriate source data are available.
6. In the conclusion, the authors note that the results are surprising in light of the known effects of GH in two extreme circumstances (acromegaly and Laron dwarfism) but do not cite the substantial literature suggesting insulin resistance in growth hormone deficient patients from other causes. Partial GH receptor blockade may more closely mimic the GH deficiency of hypopituitarism (which is often partial as well) and thus these results may not be as surprising as the authors assert.

We have read this submission. We believe that we have an appropriate level of expertise to confirm that it is of an acceptable scientific standard.

# Peer review: Referee response for version 1

**DOI:** 10.5256/f1000research.12263.r22787

**Published:** 2017-06-26

**Authors:** Laura E. Dichtel

**Affiliations:** 1 Neuroendocrine Unit, Massachusetts General Hospital and Harvard Medical School, Boston, MA, USA

The growth hormone (GH)/IGF-1 axis has a complex relationship with glucose homeostasis, as GH promotes insulin resistance while IGF-1 contributes to insulin sensitivity. Patients with acromegaly and GH excess tend to have insulin resistance that improves with GH receptor blockade (pegvisomant). The authors of this manuscript set out to investigate an interesting question—that is, would GH receptor blockade for one month in men with insulin resistance but without type 2 diabetes mellitus improve whole body insulin sensitivity (M/I) while reducing lipolysis and endogenous glucose production (EGP). Methodology in this trial was rigorous, including controlled metabolic diets the evening prior to assessment, hyperinsulinemic-euglycemic clamp with stable isotope infusions, indirect calorimetry and body composition by DXA. The authors demonstrated that despite adequate GH blockade (as evidenced by a drop in IGF-1 levels), there were no differences in insulin sensitivity, lipolysis or EGP after 1 month of GH receptor blockade. The authors thoughtfully discuss potential rationale for these negative results, including small sample size with limited power, variability of GH receptor blockade in liver versus adipose tissue as well as near-total suppression of EGP at baseline, limiting the ability to detect further suppression of EGP at follow up after pegvisomant administration. In their summary, they raise the interesting point that despite these negative results in men with insulin resistance but no diabetes, it might be worthwhile studying a more severe phenotype, such as those with untreated, newly diagnosed type 2 diabetes mellitus. This is a well written manuscript that addresses an important gap in knowledge related to the GH/IGF-1 axis and insulin resistance.

 One point for the authors to consider updating: In the abstract the subject ages are listed as 18-62 years while the methods section of the paper lists enrolled ages of 52-57 years. Given the mean age of 54.5 ± 2.1 years, I presume the latter age range is probably more accurate of subjects who were actually enrolled.

I have read this submission. I believe that I have an appropriate level of expertise to confirm that it is of an acceptable scientific standard.
